# BCL11A intellectual developmental disorder: defining the clinical spectrum and genotype-phenotype correlations

**DOI:** 10.1038/s41431-024-01701-z

**Published:** 2024-10-24

**Authors:** Angela Peron, Felice D’Arco, Kimberly A. Aldinger, Constance Smith-Hicks, Christiane Zweier, Gyri A. Gradek, Kimberley Bradbury, Andrea Accogli, Erica F. Andersen, Ping Yee Billie Au, Roberta Battini, Daniah Beleford, Lynne M. Bird, Arjan Bouman, Ange-Line Bruel, Øyvind Løvold Busk, Philippe M. Campeau, Valeria Capra, Colleen Carlston, Jenny Carmichael, Anna Chassevent, Jill Clayton-Smith, Michael J. Bamshad, Dawn L. Earl, Laurence Faivre, Christophe Philippe, Patrick Ferreira, Luitgard Graul-Neumann, Mary J. Green, Darrah Haffner, Parthiv Haldipur, Suhair Hanna, Gunnar Houge, Wendy D. Jones, Cornelia Kraus, Birgit Elisabeth Kristiansen, James Lespinasse, Karen J. Low, Sally Ann Lynch, Sofia Maia, Rong Mao, Ruta Kalinauskiene, Catherine Melver, Kimberly McDonald, Tara Montgomery, Manuela Morleo, Constance Motter, Amanda S. Openshaw, Janice Cox Palumbos, Aditi Shah Parikh, Yezmin Perilla-Young, Cynthia M. Powell, Richard Person, Megha Desai, Juliette Piard, Rolph Pfundt, Marcello Scala, Margaux Serey-Gaut, Deborah Shears, Anne Slavotinek, Mohnish Suri, Claire Turner, Tatiana Tvrdik, Karin Weiss, Ingrid M. Wentzensen, Marcella Zollino, Tzung-Chien Hsieh, C4RCD Research Group, Keri Ramsey, Telethon Undiagnosed Disease Program (TUDP), Angela Peron, Andrea Accogli, Valeria Capra, Manuela Morleo, Marcello Scala, Marcella Zollino, University of Washington Center for Mendelian Genomics (UW-CMG), Michael J. Bamshad, Dawn L. Earl, Bert B. A. de Vries, Francois Guillemot, William B. Dobyns, David Viskochil, Cristina Dias

**Affiliations:** 1 Division of Medical Genetics, Department of Pediatrics, University of Utah School of Medicine, Salt Lake City, UT USA; 2 Medical Genetics, ASST Santi Paolo e Carlo, San Paolo Hospital, Milano, Italy; 3 Department of Radiology, Great Ormond Street Hospital for Children, London, UK; 4 Department of Pediatrics, University of Washington School of Medicine, Seattle, WA USA; 5 Center for Integrative Brain Research, Seattle Children’s Research Institute, Seattle, WA USA; 6 Department of Neurology, Johns Hopkins University School of Medicine, Baltimore, MD USA; 7 Department of Neurogenetics, Kennedy Krieger Institute, Baltimore, MD USA; 8 Institute of Human Genetics, Universitätsklinikum Erlangen, Friedrich-Alexander-Universität Erlangen-Nürnberg (FAU), Erlangen, Germany; 9 Department of Human Genetics, Inselspital, Bern University Hospital, University of Bern, Bern, Switzerland; 10 Department of Medical Genetics, Haukeland University Hospital, Bergen, Norway; 11 Department of Medical Genetics, Guy’s and St. Thomas’ NHS Foundation Trust, London, UK; 12 Genomics and Clinical Genetics, IRCCS Istituto Giannina Gaslini, Genova, Italy; 13 U.O.C. Genetica Medica, IRCCS Istituto Giannina Gaslini, Genova, Italy; 14 ARUP Laboratories, Cytogenetics and Genomic Microarray, Salt Lake City, UT USA; 15 Department of Pathology, University of Utah, Salt Lake City, UT USA; 16 Department of Pediatrics, Division of Medical Genetics, Alberta Children’s Hospital Research Institute, Cumming School of Medicine, University of Calgary, Calgary, AB Canada; 17 IRCCS Fondazione Stella Maris, Pisa, Italy; 18 Dipartimento di Medicina Clinica e Sperimentale, University of Pisa, Pisa, Italy; 19 Division of Medical Genetics, Department of Pediatrics, Benioff Children’s Hospital, University of California, San Francisco, CA USA; 20 Department of Pediatrics, University of California San Diego, San Diego, CA USA; 21 Division of Genetics/Dysmorphology, Rady Children’s Hospital San Diego, San Diego, CA USA; 22 Department of Clinical Genetics, Erasmus MC University Medical Center, Rotterdam, The Netherlands; 23 INSERM UMR 1231 Equipe GAD, Université de Bourgogne, Dijon, France; 24 Unité Fonctionnelle d’Innovation diagnostique des maladies rares, FHU-TRANSLAD, CHU Dijon Bourgogne, Dijon, France; 25 Department of Medical Genetics, Telemark Hospital Trust, 3710 Skien, Norway; 26 Department of Pediatrics, CHU Sainte-Justine and University of Montreal, Montreal, QC Canada; 27 Division of Genetics and Genomics, Boston Children’s Hospital, Boston, MA USA; 28 Department of Clinical Genetics, Addenbrooke’s Hospital, Cambridge, UK; 29 Division of Evolution and Genomic Sciences School of Biological Sciences University of Manchester, Manchester, UK; 30 Manchester Centre for Genomic Medicine, St Mary’s Hospital, Manchester University NHS Foundation Trust, Manchester, UK; 31 Division of Genetic Medicine, Seattle Children’s Hospital, Seattle, WA USA; 32 University of Washington, Seattle, WA USA; 33 Centre de Référence Maladies Rares Anomalies du développement et syndromes malformatifs, Centre de Génétique, FHU-TRANSLAD, CHU Dijon Bourgogne, Dijon, France; 34 Universitätsmedizin Berlin, Institut für Medizinische Genetik und Humangenetik, Berlin, Germany; 35 Experimental Histopathology Laboratory, The Francis Crick Institute, London, UK; 36 Department of Pediatrics, Division of Pediatric Neurology, Nationwide Children’s Hospital and Ohio State University, Columbus, OH USA; 37 Department of Pediatric Immunology, Rappaport Children’s Hospital, Rambam Health Care Campus, Haifa, Israel; 38 Rappaport Faculty of Medicine, Technion - Israel Institute of Technology, Haifa, Israel; 39 North East Thames Regional Genetics Service, Great Ormond Street Hospital for Children, Great Ormond Street, London, UK; 40 Department of Neurohabilitation, Oslo University Hospital, Oslo, Norway; 41 HDR - Service de Génétique Médicale, Centre Hospitalier Métropole Savoie, Chambery, France; 42 Clinical Genetics Service, University Hospitals Bristol and Weston NHS trust, Bristol, UK; 43 Department of Clinical Genetics, Children’s Health Ireland at Crumlin, Dublin, Ireland; 44 Medical Genetics Unit, Hospital Pediátrico, Centro Hospitalar Universidade de Coimbra, Coimbra, Portugal; 45 Division of Medical Genetics, Akron Children’s Hospital, Akron, OH USA; 46 University of Mississippi Medical Center, Jackson, MS USA; 47 Northern Genetics Service, Institute of Genetic Medicine, Newcastle upon Tyne NHS Foundation Trust, Newcastle, UK; 48 Telethon Institute of Genetics and Medicine, Pozzuoli, Napoli, Italy; 49 Department of Precision Medicine, University of Campania “Luigi Vanvitelli”, Napoli, Italy; 50 Department of Genetics and Genome Sciences, Case Western Reserve University, Cleveland, OH USA; 51 Center for Human Genetics, University Hospitals Cleveland Medical Center, Cleveland, OH USA; 52 Division of Pediatric Genetics and Metabolism, University of North Carolina, Chapel Hill, NC USA; 53 GeneDx, Gaithersburg, MD USA; 54 Centre de Génétique Humaine, Université de Franche-Comté, CHU, Besançon, France; 55 Department of Human Genetics, Donders Institute for Brain, Cognition and Behavior, Radboud University Medical Center, Nijmegen, the Netherlands; 56 Pediatric Neurology and Muscular Diseases Unit, IRCCS Istituto Giannina Gaslini, Genova, Italy; 57 Centre de Recherche en Audiologie, Hôpital Necker, AP-HP. CUP, Paris, France; 58 Oxford Centre for Genomic Medicine, Oxford University Hospitals NHS Foundation Trust, Oxford, UK; 59 Nottingham Clinical Genetics Service; Nottingham University Hospitals NHS Trust, Nottingham, UK; 60 Clinical Genetics, Royal Devon and Exeter NHS Foundation Trust, Exeter, UK; 61 Department of Pathology and Laboratory Medicine, Emory University School of Medicine, Atlanta, GA USA; 62 Genetics Institute, Rambam Health Care Campus, Haifa, Israel; 63 Dipartimento Universitario Scienze della Vita e Sanità Pubblica, Sezione di Medicina Genomica, Università Cattolica Sacro Cuore, Roma, Italy; 64 Genetica Medica, Fondazione Policlinico Universitario A. Gemelli IRCCS, Roma, Italy; 65 Institute for Genomic Statistics and Bioinformatics, University Hospital Bonn, Rheinische Friedrich-Wilhelms-Universität Bonn, Bonn, Germany; 66 Neural Stem Cell Biology Laboratory, The Francis Crick Institute, London, UK; 67 Department of Medical & Molecular Genetics, School of Basic and Medical Biosciences, Faculty of Life Sciences & Medicine, King’s College London, London, UK; 68 Center for Rare Childhood Disorders, Translational Genomics Research Institute, Phoenix, AZ USA; 69 Telethon Undiagnosed Diseases Program. Telethon Institute of Genetics and Medicine, Pozzuoli, Napoli Italy; 70 Present Address: Department of Experimental and Clinical Biomedical Sciences, Università degli Studi di Firenze, Firenze, Italy; 71 Present Address: Medical Genetics, Meyer Children’s Hospital IRCCS, Firenze, Italy; 72 Present Address: Wessex Regional Genetics Service, Princess Anne Hospital, Southampton, UK; 73 Present Address: Department of Pediatrics and Physiology & Membrane Biology, University of California, Davis, CA USA; 74 Present Address: Department of Medical Genetics, Guy’s and St. Thomas’ NHS Foundation Trust, London, UK; 75 Present Address: Division of Human Genetics, Cincinnati Children’s Hospital, and Department of Pediatrics, College of Medicine, University of Cincinnati, Cincinnati, OH USA; 76 Present Address: Division of Genetics and Metabolism, Department of Pediatrics, University of Minnesota, Minneapolis, MN USA

**Keywords:** Neurodevelopmental disorders, Disease genetics, Genotype, Genetic counselling, Neurodevelopmental disorders

## Abstract

An increasing number of individuals with intellectual developmental disorder (IDD) and heterozygous variants in *BCL11A* are identified, yet our knowledge of manifestations and mutational spectrum is lacking. To address this, we performed detailed analysis of 42 individuals with *BCL11A*-related IDD (BCL11A-IDD, a.k.a. Dias-Logan syndrome) ascertained through an international collaborative network, and reviewed 35 additional previously reported patients. Analysis of 77 affected individuals identified 60 unique disease-causing variants (30 frameshift, 7 missense, 6 splice-site, 17 stop-gain) and 8 unique *BCL11A* microdeletions. We define the most prevalent features of BCL11A-IDD: IDD, postnatal-onset microcephaly, hypotonia, behavioral abnormalities, autism spectrum disorder, and persistence of fetal hemoglobin (HbF), and identify autonomic dysregulation as new feature. BCL11A-IDD is distinguished from 2p16 microdeletion syndrome, which has a higher incidence of congenital anomalies. Our results underscore BCL11A as an important transcription factor in human hindbrain development, identifying a previously underrecognized phenotype of a small brainstem with a reduced pons/medulla ratio. Genotype-phenotype correlation revealed an isoform-dependent trend in severity of truncating variants: those affecting all isoforms are associated with higher frequency of hypotonia, and those affecting the long (BCL11A-L) and extra-long (-XL) isoforms, sparing the short (-S), are associated with higher frequency of postnatal microcephaly. With the largest international cohort to date, this study highlights persistence of fetal hemoglobin as a consistent biomarker and hindbrain abnormalities as a common feature. It contributes significantly to our understanding of BCL11A-IDD through an extensive unbiased multi-center assessment, providing valuable insights for diagnosis, management and counselling, and into BCL11A’s role in brain development.

## Introduction

BCL11A-related Intellectual Developmental Disorder (BCL11A-IDD), also known as intellectual developmental disorder with persistence of fetal hemoglobin [OMIM #617101] or Dias-Logan syndrome, is an autosomal dominant condition caused by heterozygous pathogenic variants in *BCL11A* (HGNC:13221) [1, 2]. *BCL11A* encodes a Krüppel-like sequence-specific C2H2 zinc finger (ZnF) transcription factor required for the fetal-to-adult hemoglobin transition via transcriptional repression [3]. BCL11A is highly expressed in the developing mammalian brain, regulating multiple developmental processes, including subtype identity in deep-layer projection neurons, differentiation and thalamocortical integration of layer IV projection neurons, cell-polarity switch and radial migration of upper-layer projection neurons [2, 4, 5]. We have previously shown that *Bcl11a* haploinsufficiency in mice leads to impaired memory and social behavior, along with regionalized reduction of brain size, including of the hippocampus, corpus callosum and cerebellum (superior vermis) [2].

The significance of BCL11A in human brain development is underscored by the identification of individuals with neurodevelopmental phenotypes associated with pathogenic heterozygous variants or copy number loss of *BCL11A* [2, 6–8]. BCL11A-IDD has been identified in individuals with heterozygous loss-of-function variants [2, 9–13] and N-terminus missense variants acting as hypomorphic alleles, impacting *BCL11A* transcriptional activity, subcellular localization [2] and increasing proteasomal degradation of the BCL11A protein [14]. Despite these findings, a detailed understanding of the phenotypic spectrum of BCL11A-IDD is lacking, as is the effect of genotypic variation on disease features and severity. Moreover, incomplete annotation of human and mouse BCL11A isoforms (www.ensembl.org) poses challenges in determining isoform-specific tissue and developmental expression. Experimental evidence supports the expression of three main BCL11A isoforms in developing human brain, previously (and herein) designated BCL11A-XL (extra-long, 835 aa), BCL11A-L (long, 773 aa), and BCL11A-S (short, 243 aa) [15].

To address these knowledge gaps, we aimed to define the phenotypic spectrum of BCL11A-IDD and determine genotype-phenotype correlations by analyzing a large cohort of affected individuals. Here we present detailed phenotypic data on 42 participants with *BCL11A* variants consistent with BCL11A-IDD and review an additional 35 reported patients. We evaluate the effect of variants in the context of different *BCL11A* isoforms and identify a putative isoform-specific genotype-phenotype effect. We demonstrate a broad spectrum of severity of neurodevelopmental phenotypes and mild dysmorphic features, and confirm elevated hemoglobin F (HbF) as a hallmark of the condition. We provide further evidence for hindbrain abnormalities and report novel rare autonomic phenotypes.

## Subjects and methods

### Subjects

Present cohort: individuals with BCL11A-IDD were identified through clinicians’ direct clinical practice, the Deciphering Developmental Disorders Study [7], GeneMatcher [16] and publicly available databases (Decipher [17] and ClinVar [18]). All individuals had a previously identified likely pathogenic or pathogenic variant in *BCL11A*. Individuals previously reported by co-authors are included with updated phenotypic information: P5 [19], P8 and P25 [20], P30, P36, P37 [2]. Informed consent for participating in diagnostic or research studies had been obtained according to institutional review boards and local regulatory authorities (see Supplementary Material). Written consent was obtained for sharing of deidentified clinical information, and photographs, where applicable, according to principles outlined in the Declaration of Helsinki. Participants did not receive a stipend.

Additional previously reported individuals were identified via literature search in PubMed (to April 5, 2022). The following search terms were used: “BCL11A”, “Dias-Logan syndrome”, “2p16.1 deletion”. For individuals with copy number variants (CNVs), only those encompassing *BCL11A* alone or *BCL11A* and non-coding genes were included. Individuals with larger genomic deletions encompassing additional protein-coding genes were excluded from this cohort and considered only as a comparison group of selected phenotypes (reported to July 1, 2021) [8, 21].

For DECIPHER (April 17, 2021) and ClinVar (Oct 2023), where the submitter was unavailable or unable to provide further clinical information, open access pathogenic and likely pathogenic variants with at least one associated phenotypic term are presented as aggregate data.

### Phenotyping

Clinicians provided retrospective clinical and molecular data on a standardized proforma. Facial features of available photographs were assessed independently by two experienced clinical geneticists (A.P. and C.D.) and a final consensus phenotype was recorded using The Elements of Morphology: Standard Terminology nomenclature [22]. Clinical manifestations were annotated using Human Phenotype Ontology (HPO) (Supplementary Table 1) [23]. Frequencies of clinical manifestations were calculated using the number of individuals for whom evidence of presence/absence of each manifestation was available. Age and sex-adjusted standard deviations (SD) for growth parameters recorded as raw numbers were calculated with the UKWHO reference in the childsds package version 0.7.6 [24] in R v.4.0.3. Percentiles/SD provided by the clinician were used where primary measurements were unavailable.

### Variant annotation

**Fig. 1:**
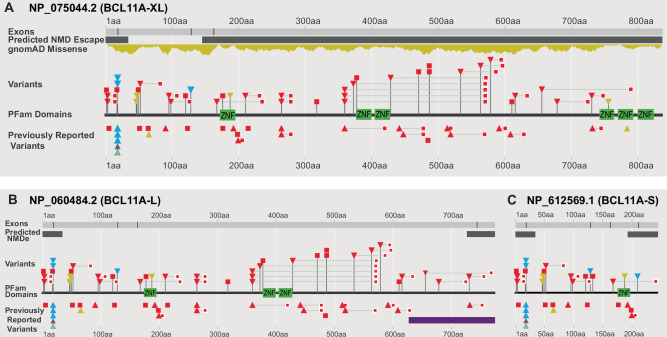
BCL11A genomic variants. BCL11A genomic variants annotated to MANE select BCL11A-XL (**A**), BCL11A-L (**B**) and BCL11A-S (**C**) isoforms. Stop gain: red square; frameshift: red triangle, with downstream premature termination codon as small red square; missense variants: yellow triangle; splice variants: light blue triangle. Light blue outline on red triangle: frameshift and potential splice variant ClinVar_VCV000987092.1; light blue outline on yellow triangle: missense and potential splice variant ClinVar_VCV000987093.1 (see Supplementary Table 2). Green: C2H2 DNA binding zinc finger domains. Dark gray bars: regions of predicted NMD escape by “start proximal” and “last exon junction” mechanisms. Purple bar: region of putative interaction with TBR1 (den Hoed et al. [37]). This figure was generated in collaboration with the Decipher team (https://deciphergenomics.org).

Variants annotated to GRCh37 were mapped to GRCh38 using Assembly Converter (Ensembl release 103 - February 2021). CNVs were visualized as a custom track in Ensembl (GRCh38.p13). Three *BCL11A* transcript isoforms with evidence of expression in brain tissue [15] were selected for analysis: *BCL11A*-XL (NM_022893.4, MANE transcript), *BCL11A*-L (NM_018014.4) and *BCL11A*-S (NM_138559.2) (Fig. 1). Variants were classified based on predicted effects on these isoforms and nonsense mediated decay (NMD) [25] (Supplementary Table 2): *PTVa*, premature termination codon (PTC) in all 3 isoforms; *PTVb*, PTC affects only BCL11A-XL and -L; *MISS*, missense variants; *SPL*, splice variants predicted to alter the reading frame; *CNV*, copy number variants (microdeletions).

### Fetal forebrain and cerebellum samples

Human fetal hindbrain samples were provided by: 16 and 18 post-conception weeks [pcw], the Joint MRC/Wellcome Trust (MR/R006237/1) Human Developmental Biology Resource (HDBR,) with ethical approval (RECs 18/NE/0290 and 18/LO/0822, www.hdbr.org); 12 pcw, the Birth Defects Research Laboratory (BDRL), University of Washington, Seattle, WA, with ethics board approval, and in accordance with ethical and legal guidelines of Seattle Children’s Hospital Institutional Review Board. Immunohistochemistry was performed using standard methods detailed in Supplementary Material.

### Neuroimaging

Retrospectively acquired brain MRI scans of 13 individuals were analyzed by an experienced pediatric neuroradiologist (F.D’A.), blinded to genotype. Posterior fossa abnormalities were assessed qualitatively and using quantitative measurements when DICOM images were available: the vermian height, vermian antero-posterior diameter, antero-posterior midbrain-pons junction and antero-posterior mid-pons diameter were measured on the sagittal midline and compared with normal values for age-matched controls available in literature [26]. Values below the 3^rd^ percentile were considered abnormal. A cranio-caudal ratio with midbrain and/or medulla oblongata less than 1.5:1 was used to define an abnormal pons [27].

### Statistical analysis

Categorical variables are presented as n (frequency, %), and continuous variables as median with ranges. Statistical analyses were performed using Graphpad Prism v.8.0.1. Graphical representations were generated using Graphpad Prism and R v.4.0.3 (ggplot2 v.3.3.3, circlize v.0.4.13). For statistical testing, despite standardized data recording on the same proforma, we recognize we cannot correct for all the technical variation in prior assessment of individuals. We therefore performed descriptive statistics and where possible applied unpaired t-test and Fisher’s exact test (FET) for comparing frequencies between groups, at a significance threshold of *p* < *0.05*.

## Results

### Cohort overview

We present 42 individuals (18 female, 24 male) with heterozygous pathogenic or likely pathogenic variants in *BCL11A* including 35 previously unreported in a publication and seven previously reported with updated and detailed clinical assessments. Median age at molecular diagnosis was 8^+11/12^ years (range 1–41 y); median age at last assessment was 9^+2/12^ years (range 2–42 y) (Supplementary Table 1). We analyzed data from 17 additional previously published cases and 18 records deposited in publicly available databases (not included in our 42-patient cohort), for a total of 77 patients with BCL11A-IDD (“combined dataset”, *n* = 77, present cohort combined with previously reported and aggregate data from open access databases) [2, 9–13]. The main clinical manifestations of each subgroup and of the combined dataset are presented in Table 1, with additional details provided in Supplementary Table 1. To address potential biases in data ascertainment, results of the “present cohort” (*n* = 42) and of the “combined dataset” are presented separately.

**Table 1:** Demographic and clinical information about individuals with pathogenic and likely pathogenic variants in *BCL11A*.

Phenotype		Present cohort (%)		Previously published patients	Publicly available data^a^	Total (%)		
Total number of patients		42		17	18	77		
Sex	Females	18/42	(43%)	8/17	3/6	29/65	**(45%)**	
	Males	24/42	(57%)	9/17	3/6	36/65	**(55%)**	
Prenatal and birth history								
Abnormal prenatal history		13/38	(34%)	2/7	1/1	16/46	**(35%)**	
Congenital malformations		6/41	(15%)	1/17	4/7	11/65	**(17%)**	
Growth parameters								
Congenital microcephaly^b^		1/15	(7%)	0/5	1/1	2/21	**(10%)**	
Post-natal microcephaly^b^		16/35	(46%)	8/14	2/3	26/52	**(50%)**	
Median height SD (*n*, range)		−0.47 (*n* = 34; −2.80 - +1.30)		−0.28 (*n* = 9; −3.24 - +2.01)	NA	−0.45 (*n* = 43; −3.24 - +2.01)		
Median weight SD (*n*, range)		−0.42 (*n* = 33; −2.75 - +2.24)		0.14 (*n* = 7; −2.00 - +1.24)	NA	−0.32 (*n* = 40; −2.75 - +2.24)		
Neurodevelopment								
DD/IDD		38/40	(95%)	16/16	16/16	70/72	**(97%)**	
Mild		7/38	(18%)	1/16	1/16	9/70	**(13%)**	
Moderate		14/38	(37%)	7/16	3/16	24/70	**(34%)**	
Severe/profound		7/38	(18%)	4/16	1/16	12/70	**(17%)**	
Unknown severity		10/38	(26%)	4/16	11/16	25/70	**(36%)**	
Delayed speech dev.		36/39	(92%)	16/16	3/3	55/58	**(95%)**	
Delayed gross motor dev.		37/39	(95%)	13/13	NA	50/52	**(96%)**	
Delayed fine motor dev.		24/26	(92%)	4/4	NA	28/30	**(93%)**	
Behavior								
Autism Spectrum Disorder		14/37	(38%)	4/13	1/1	19/51	**(37%)**	
Other behavioral abn.^c^		27/39	(69%)	6/10	1/1	34/50	**(68%)**	
Seizures		8/37	(22%)	4/15	1/1	13/53	**(25%)**	
Median age at seizure onset (n, range)		4.25 (*n* = 8; 5m–10y)		3 (*n* = 3; 2m–3.3 y)	NA	3.3 (*n* = 11; 2m–10y)		
Hypotonia		25/38	(66%)	6/7	4/4	35/49	**(71%)**	
Abnormal motor function^d^		13/31	(42%)	2/2	4/4	19/37		**(51%)**
Abnormal brain MRI		21/38	(55%)	7/10	1/1	29/49	**(59%)**	
Posterior fossa abn.		12/38	(32%)	4/10	1/1	17/49	**(35%)**	
Brainstem abn.		12/37	(32%)	3/10	0/1	15/48	**(31%)**	
Corpus callosum abn.		7/38	(18%)	2/10	NA	9/48	**(19%)**	
Cortical malformation		2/38	(5%)	0/10	NA	2/48	**(4%)**	
Craniofacial features								
Epicanthus		12/41	(29%)	6/11	3/3	21/55	**(38%)**	
Wide nose		21/41	(51%)	5/11	NA	26/52	**(50%)**	
Malar flattening		21/41	(51%)	8/11	1/1	30/53	**(57%)**	
Full cheeks		19/41	(46%)	3/11	NA	22/52	**(42%)**	
Thin vermillion upper lip		14/41	(34%)	8/12	1/1	23/54	**(43%)**	
Thick/everted lower lip		22/41	(54%)	7/11	1/1	30/53	**(57%)**	
Cleft/high palate		6/41	(15%)	3/11	NA	9/52	**(17%)**	
Ear abnormalities		24/41	(59%)	9/11	NA	33/52	**(64%)**	
Other								
Strabismus		21/35	(60%)	9/15	NA	30/50	**(60%)**	
Scoliosis		6/30	(20%)	2/2	NA	8/32		**(25%)**
Joint hypermobility		12/33	(36%)	7/15	NA	19/48	**(40%)**	
Autonomic features^e^		5/30	(17%)	NA	NA	5/30		**(17%)**
Constipation		11/31	(35%)	2/2	NA	13/33		**(39%)**
Abn. immune system		1/19	(5%)	NA	NA	1/19	**(5%)**	
Increased HbF		23/23	(100%)	9/9	NA	32/32	**(100%)**	

The percentages in bold are the total frequencies of each row.

HPO terms for each clinical manifestation can be found in Supplementary eTable 1.

*NA* Not Available, *SD* Standard Deviation, *DD* developmental delays, *IDD* intellectual developmental disorder, *dev.* development, *abn.* abnormalities, *m* months, *y* years, *MRI* magnetic resonance imaging, *HbF* fetal hemoglobin.

^a^From DECIPHER and ClinVar.

^b^Includes all patients where microcephaly/normal is reported regardless of whether measured values are presented.

^c^See main text for detail.

^d^Ataxia, broad based gait, or spastic paraplegia.

^e^Peripheral vascular signs, reduced or increased sensitivity to pain, nocturia and other urinary disturbance, abnormal sweating.

### Mutational spectrum

The present cohort of 42 includes 32 individuals with 27 unique protein truncating variants (PTV: 11 individuals heterozygotes for 10 unique PTVa variants, 21 for 17 unique PTVb variants); 3 splice variants (SPL); 4 missense variants (MISS) (Fig. 1, Supplementary Table 3); and 3 with unique CNVs (Supplementary Fig. 1A, Supplementary Table 4). Notably, only 5 variants are predicted to efficiently undergo nonsense mediated decay (NMD) in all 3 isoforms (PTVa2). All others are predicted to escape or have reduced efficiency of NMD via different mechanisms, leading to truncated proteins with or without changes to the open reading frame (Supplementary Table 3). Overall, the combined dataset includes 56 patients with PTV (21 PTVa and 35 PTVb), 6 with SPL, 7 MISS and 8 CNV.

We report 2 novel missense variants, His188Arg and Asn756Lys, classified as “pathogenic” by AlphaMissense [28]. His188Arg localizes to the first C2H2 ZnF present in all 3 major isoforms (Fig. 1). Asn756Lys locates to the fourth BCL11A-XL C2H2 ZnF in the C-terminus ZnF cluster required for globin repression [3]. Based on previously reported crystal structure of BCL11A [29], we computationally predicted the Asn756Lys variant alters the zinc finger structure (Supplementary Fig. 1B) .

Inheritance information was available for 40 individuals. Thirty-three (33/40, 83%) had de novo variants. Parental germline mosaicism is suspected in two sib pairs from unaffected parents in 2 families (P2 and P3; P23 and P24); parents do not carry the variant in the tissue tested (buccal (parents of P2,3) and blood (parents of P23,24)) and do not present cognitive or neurobehavioral phenotypes. In another affected individual, the variant was not maternally inherited (P11; paternal DNA unavailable).

Frameshift variants were inherited in two families (P31, P32; neither parent formally assessed). P31’s heterozygote mother was described as having learning difficulties. P32’s heterozygote father was described as having “autistic traits” and mild cognitive impairment; parental ratio of mutant and wildtype allele could not confirm mosaicism (48.7% ALT/REF in blood). P32’s variant, Ser657ThrfsTer134, is expected to generate a truncated protein with an altered amino acid sequence disrupting the C-terminus C2H2 ZnF; frameshifted *BCL11A-XL* cDNA is detected in lymphoblastoid cells (Supplementary Fig. 1C; Supplementary Methods). However, *BCL11A* expression in lymphoblastoid cells of P32 was not decreased in comparison to his mother without the variant (Supplementary Fig. 1D, E), consistent with NMD escape.

### Neurodevelopmental phenotypes

Developmental delay and/or intellectual disability (ID) was present in all individuals in the present cohort, except two (38/40, 95%). The severity ranged from mild to severe/profound, with the majority having moderate ID (Table 1, Supplementary Fig. 2A). Interestingly, two individuals with PTVb class variants (P16, Ser378Ter; P32, Ser657ThrfsTer134) had a normal cognitive function assessment, with IQ scores of 93 and 80, respectively. Both were diagnosed with autism spectrum disorder (ASD), with P16 diagnosed with dyslexia and dysgraphia, and P32 receiving speech therapy and special education.

Speech and language were affected in most individuals (Table 1), involving both receptive and expressive language. Median age at first words was 2 years (range 9 m - 4^+6/12^y). Eight had no speech at last assessment. Dysarthria, articulation problems and/or speech apraxia were reported in 7. Gross and fine motor development delay were common, with a median age of 11 months for independent sitting (range 6–19 months) and 29 months for walking independently or with support (range 14 months – 9 years).

ASD was diagnosed in 38% (14/37), with a similar prevalence in the combined datasets: 19/51, 37%. Prevalence of ASD was greater, albeit non-significant (FET, *p* = 0.6942), in patients with PTVb (12/14) vs. PTVa (4/10) variants. Additional behavioral problems were reported in 69% of patients (27/39), including aggressiveness (*n* = 13), repetitive behavior (*n* = 13), sleep disturbances (*n* = 11), attention deficit hyperactivity disorder (*n* = 10), and anxiety (*n* = 13), some co-occurring in the same individual (Supplementary Table 1).

### Additional neurologic manifestations

Hypotonia was observed in 25/38 patients (66%) and was significantly more frequent in individuals with PTVa variants (10/11, 91%) when compared to MISS (1/4, 25%, FET, *p* = *0.0330*). Twenty-two percent of patients (8/37) had seizures, with median age at onset of 4^+3/12^ years (range 5 months - 10 years). Two additional individuals had suffered a single seizure and 3 had abnormal EEG findings without clinical seizures. Seizures were polymorphic, including generalized tonic-clonic, myoclonic, and focal-onset, with no dominant seizure type (Supplementary Table 1). Motor impairment, such as ataxia, broad-based gait, or lower limb spasticity, was reported in 13/31 individuals (42%).

### Hindbrain abnormalities are common in BCL11A-IDD

**Fig. 2:**
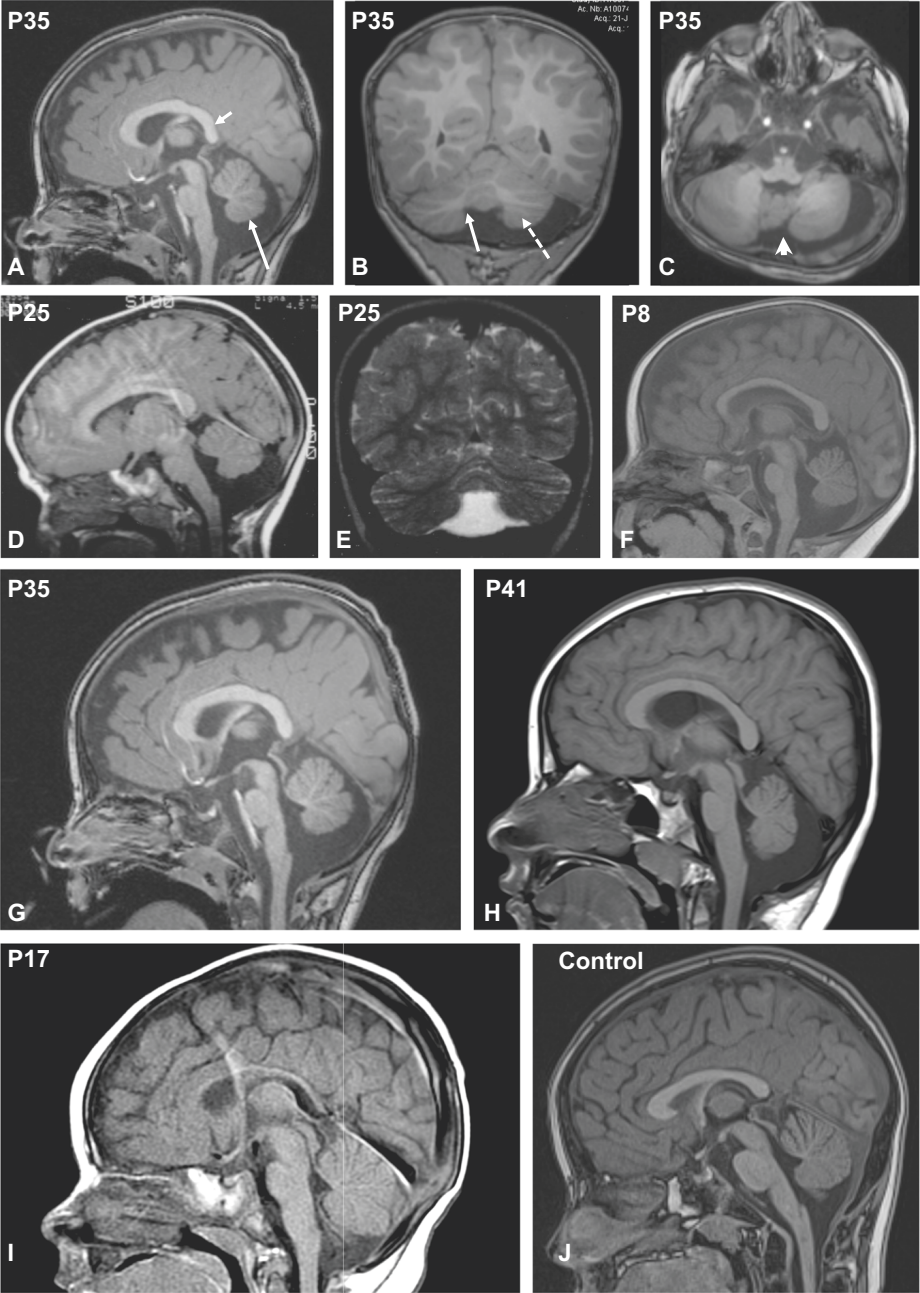
Neuroradiological features of individuals with BCL11A-IDD. Sagittal (**A**), Coronal (**B**) and axial (**C**) 3D T1 weighted-images (WI) in P35 showing inferior vermian hypoplasia (long arrows in **A** and **B**) with associated enlargement of the tegmento-vermian, mild reduction in size of the posterior aspect of the corpus callosum (short arrow in **A**), hypoplasia of the left cerebellar hemisphere (dashed arrow in **B**) and mild dysplasia of the vermis (arrowhead in **C**). Sagittal T1 WI (**D**) and coronal T2 WI (**E**) in P25 and sagittal T1 WI in P8 (**F**) showing isolated vermian hypoplasia with associated enlargement of the tegmento-vermian. Vermian hypoplasia was confirmed with measurements compared to normal values described in Jandeaux et al. [26]. The pons is small in both patients. Sagittal T1 WI in P35 (**G**), P41 (**H**), and P17 (**I**) showing the short pons and abnormally elongated medulla. Normal control for comparison on the bottom right (**J**).

**Fig. 3:**
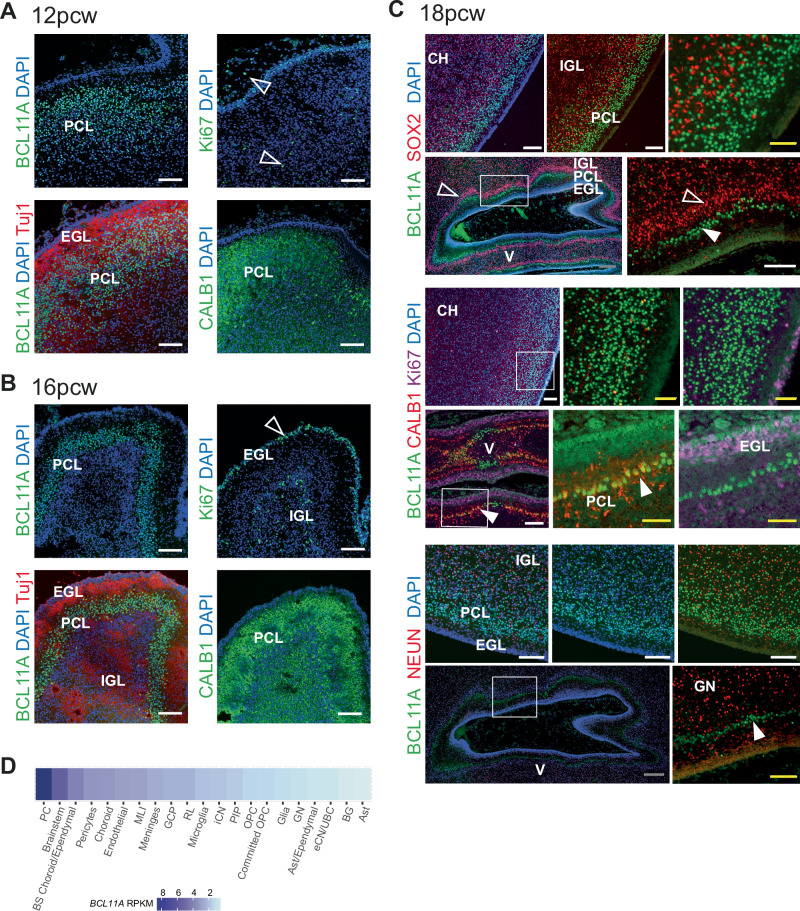
BCL11A expression in developing human cerebellum. Immunohistochemistry of human fetal cerebellum at 12 (**A**), 16 (**B**) and 18 (**C**) weeks post conception. CH cerebellar hemisphere, V developing vermis (with island like fissures); filled arrow, Purkinje cells; open arrow, cerebellar granule neuron precursors; EGL external granule layer, PCL Purkinje cell layer, IGL internal granule layer, GN granule neurons. Bars: gray, 200 µm; white, 100 µm; yellow, 50 µm. **D** BCL11A normalized gene expression by cell type in developing human cerebellum (data from Aldinger et al. [30]). RPKM, reads per kilobase of transcript per million mapped reads.

Brain abnormalities were identified on MRI in 55% of scanned patients (21/38) and 59% in the combined dataset (29/49, Supplementary Table 1, Supplementary Fig. 2B). The most common findings in our cohort were cerebellar abnormalities (12/38, 32%) and brainstem abnormalities (12/37, 32%) (Fig. 2). A small or hypoplastic cerebellar vermis was observed in all of these (Supplementary Table 5). No differences per variant type were detected. BCL11A (a.k.a. CTIP1) is highly expressed in Purkinje cells (PC) of the rodent cerebellum [30, 31]. Previous single cell gene expression analysis showed high *BCL11A* expression in human cerebellum PCs, [30] yet protein expression beyond Carnegie stage 19 in human development has not been investigated. Thus, we further explored expression in human tissues using immunohistochemistry, confirming high protein expression in the cerebellar PC layer during human development from 12 to 18 pcw (Fig. 3A–C).

The primary brainstem imaging finding was a small pons, objectively and/or relative to medulla, consistent with *BLC11A* expression in the developing human brainstem [30] (Fig. 3D). Supratentorial abnormalities were rare, occurring in only 2 patients (Supplementary Table 5). Seven of 38 patients (18%) had callosal abnormalities, a frequency comparable to that observed in reported large CNVs (3/16, 19%).

We compared the frequency of brain malformations in the combined dataset of BCL11A-IDD with that in patients reported with large contiguous gene deletions encompassing *BCL11A* (Supplementary Table 6). Cortical malformations are significantly less frequent (FET, *p* = *0.0109*) in individuals with variants or deletions affecting *BCL11A* only (2/38, 5%) compared with those carrying large CNVs (5/17, 29%) (Supplementary Fig. 2B).

### Craniofacial phenotype

**Fig. 4:**
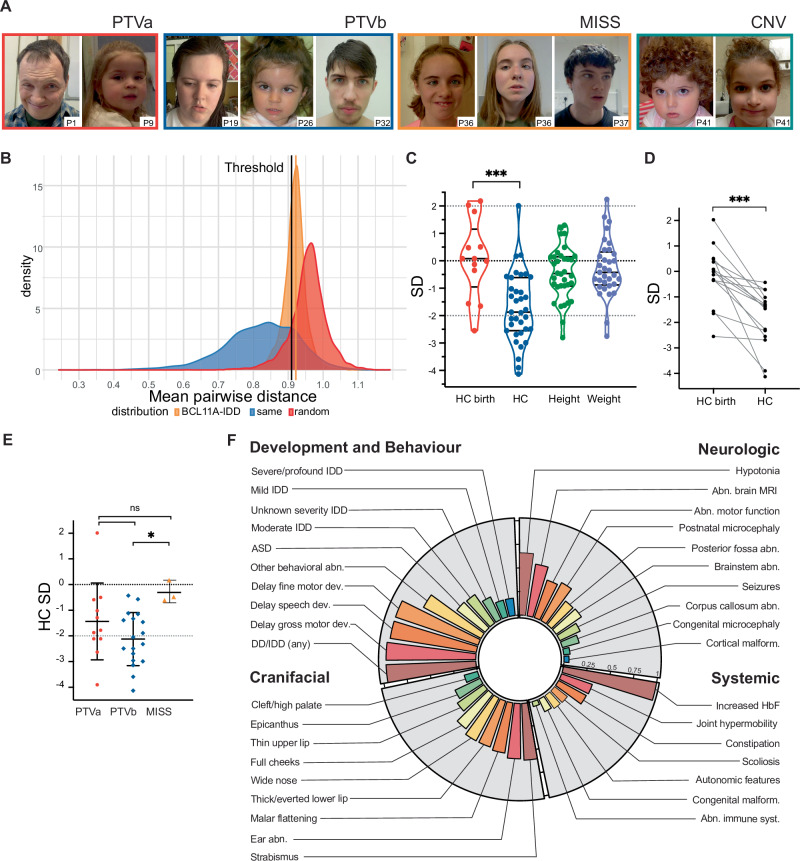
Physical features of individuals with BCL11A-IDD in the present cohort. **A** Facial features of selected individuals with *BCL11A* pathogenic variants, grouped according to variant class (see methods). Patient number is indicated in the bottom right of each image. Approximate ages at time of photographs (y, years; m, months): P1, 42 y; P9, 3y2m; P19, 16 y; P26, 4y6m; P32, 18 y; P36, 11y2m (left), 12y10m (right); P37, 16 y; P41 3y3m (left), 7y8m (right). Additional photographs of the face (profile) and limbs are available in Supplementary Fig. 2. PTV: Protein Truncating Variants (PTVa, type a; PTVb: type b); MISS: missense variants; CNV: Copy Number Variants. No photographs are available for patients with splice-site variants. **B** GestaltMatcher comparison of the distance distribution among BCL11A-IDD individuals (orange), the random selection from the subjects with 328 disorders (red), and the selection with the same disorder (blue). The black vertical line is the threshold that classifies whether it is the same disorder or random selection. 34.5% of the BCL11A distribution is below the threshold, indicating only a small portion of BCL11A individuals presenting similar facial gestalt. **C** Distribution of growth parameters (standard deviations, SD) of present cohort (a single value for each individual is represented); lines indicate median, upper and lower quartiles; *** unpaired t-test *p* = 0.0001. *n* = 13 HC birth; *n* = 35 HC; *n* = 34 height; *n* = 33 weight. **D** Head circumference (HC) at birth and postnatal (SD for age and sex) for individuals in present cohort and previously reported where both measurements available; *n* = 15 (13 PTV, 1 SPL (splice), 1 CNV); paired t-test: ***p < 0.0001. **E** Head circumference SDs for individuals in present cohort (PTVa, *n* = 11; PTVb, *n* = 18; MISS, *n* = 3); lines indicate median, upper and lower quartiles; unpaired t test: *p = 0.0083; ns not significant. **F** Circular bar plot representing frequencies for phenotypic features in the present cohort; bars are proportional to frequency, 25% intervals indicated by ticks. DD developmental delays, IDD intellectual developmental disorder, ASD autism spectrum disorder, abn. abnormalities, MRI magnetic resonance imaging, HbF fetal hemoglobin.

Mild dysmorphisms were shared among patients regardless of variant class, however we did not identify a uniquely recognizable gestalt (Fig. 4A; Table 1). We tested this using GestaltMatcher [32, 33] for facial features analysis (see Supplementary Materials), revealing the absence of a specific facial gestalt in BCL11A-IDD individuals (Fig. 4B), although the individuals below age ten years exhibited relatively higher similarities (Supplementary Fig. 3A). Furthermore, no facial similarities linked to specific variant types were observed (Supplementary Fig. 3B).

At the feature-specific level, common craniofacial features included epicanthus (12/41, 29%), wide nose (21/41, 51%), malar flattening (21/41, 51%), full cheeks (19/41, 46%), thin upper lip vermillion (14/41, 34%), thick or everted vermillion of the lower lip (22/41, 54%), and external ear abnormalities (24/41, 59%) (Supplementary Table 1). Facial appearance changed with age (where longitudinal information was available), with a round face with full cheeks and thick/everted vermillion of the lower lip more evident in younger ages, in keeping with higher phenotypic similarity below age 10 (Supplementary Fig. 3A), and a less specific longer face in adolescence and young adulthood. Ear abnormalities varied, most commonly small and/or attached earlobes. Sixty percent of individuals had strabismus (21/35). Increased body hair (synophrys, thick eyebrows, long eyelashes, or hypertrichosis) was recorded in 11/41 (27%). Supplementary Fig. 2D and Supplementary Table 1 display additional craniofacial features.

### Prenatal and birth

Abnormal prenatal history was reported in 34% of cases (13/38, detailed in Supplementary Table 1). Postnatally, congenital malformations were observed in 6/41 patients (15%), including polydactyly, cleft palate, pulmonary artery branch stenosis, craniosynostosis, and umbilical hernia. The frequency of congenital abnormalities in the present cohort was significantly lower than in reported individuals with large CNVs encompassing additional coding genes (Supplementary Table 6, 12/20; *p* = *0.0006*). This difference remained significant with the combined dataset (*p* = *0.0004*).

### Growth

Head circumference (HC) was normal at birth in all patients with data available, except one with congenital microcephaly (P29). Median birth HC SD was 0.08 (range −2.55 to 2.18 SD). Postnatal microcephaly (below −2SD or 3^rd^ centile) was observed in 16/35 (46%, Table 1; Fig. 4C). Postnatally, HC for age was significantly smaller than at birth (median SD −1.87 vs 0.08, *p* < *0.0001*) (Fig. 4C), with progressive relative postnatal microcephaly in most patients with consecutive measurements (*p* < *0.0001*, Fig. 4D). Individuals with PTVb class variants had overall smaller head size within the cohort (median SD −2.124), statistically significantly smaller compared to those with MISS variants (*p* = *0.0083*; Fig. 4E).

Most patients had normal height and weight for age (Supplementary Table 1); only 2 had stature below −2SD. Three individuals were reported to have short stature, though measurements were unavailable. Median height and weight SDs were −0.47 and −0.42, respectively (Fig. 4C) and were not significantly different per variant class (Supplementary Fig. 2G).

### Hematological phenotype

All 23 individuals in whom quantification was performed had HbF levels above maximum reference for age (ref. of <2% from age 2 years; before 2 years HbF may be physiologically elevated). With a median age at last HbF measurement of 10.5 years (range 5–41), the median value of HbF was 15.6% (range 4.1–35), with an inverse trend between age and HbF (Supplementary Fig. 2C) and no detectable correlation with variant class. In individuals with sequential measurements, HbF decreased over time, yet remained above reference levels for age (Supplementary Table 7).

### Additional features

Scoliosis was noted in 20% of affected individuals (6/30), including in 5/11 patients over 10 years of age. Joint hypermobility/laxity was reported in 36% of individuals (12/33). Constipation was reported in 35% (11/31). Signs of autonomic dysfunction in 5/30 (17%) consisted mostly of cutis marmorata, intermittently cold extremities or Raynaud’s phenomenon, and altered sensitivity to pain (Supplementary Fig. 2H).

Only one individual was identified with abnormalities of the immune system (P39, Supplementary Table 1). In four other patients where an immunoglobulin panel was performed, this was normal. Malignancies were queried because somatic variants in *BCL11A* are reported in sporadic malignant tumors, however no individuals had been diagnosed with solid or hematologic malignancies. One patient (P41) had a benign tumor (osteochondroma).

## Discussion

We present the comprehensive phenotypic and genotypic spectrum of BCL11A*-*IDD based on 77 affected individuals: 42 from our cohort (Fig. 4F) and 35 from previous reports, highlighting persistence of fetal hemoglobin as constant and hindbrain abnormalities as common features. While recurrent subtle dysmorphisms were observed, BCL11A-IDD is not associated with an objectively distinct facial appearance. We observed a trend towards a more severe phenotype for protein truncating variants (PTV) compared to missense and splice variants. PTVb variants, sparing the BCL11A-S isoform, tended to exhibit more severe neurobehavioral features, while PTVa variants trended toward a higher frequency of hypotonia, though larger cohorts are needed for further confirmation. HbF persistence in all missense variant patients tested is consistent with loss of transcriptional repression, through reduced activity or proteasomal degradation of mutant protein [14].

Intrafamilial variability, reduced penetrance, or undetected parental mosaicism may explain affected children with variants inherited from apparently un- or mildly affected heterozygote parents in two families. We also report the first two cases of apparent germline mosaicism in BCL11A-IDD, specifically 2 in 35 families where parents tested negative, i.e., ~6%, or 4% (2 in 50) considering the combined cases with parental testing. Given the variability of neurobehavioral and cognitive phenotypes, we recommend parental testing and detailed assessment of parental phenotypes to ensure appropriate recurrence risks and genetic counselling. We recommend quoting a ~4% recurrence risk for parents of a single affected child until larger cohorts are identified.

The broad variability of neurodevelopmental phenotypes and broad range of achievement of developmental milestones in individuals with pathogenic variants in *BCL11A*, even for the same variant class (Supplementary Table 1) is highlighted by our extensive cohort, which includes patients assessed independently in different centers, thus reducing biases. It is likely that additional genomic variation and non-genetic factors influence this variability. Further studies in extended cohorts will be required to confidently ascertain penetrance and identify modifiers [34].

Most variants may escape NMD (Supplementary Table 3) by different mechanisms [25], resulting in significantly truncated proteins, with or without aberrant protein sequences. Using allele-specific expression, Teran et al. [35] predictively modeled that rare variants (MAF < 0.001%) are less likely to escape NMD [35]. Variants classified as pathogenic in ClinVar were more likely to exhibit allelic imbalance across tissue types due to NMD. We could not confirm this in P32 with the methods used. The gnomAD database (v4.0) [36], records 25 heterozygous *BCL11A* variants potentially truncating at least one of the three major isoforms discussed here, excluding those annotated as “pathogenic” (Supplementary Table 8). All are anticipated to escape NMD. gnomAD includes six alleles disrupting the same leucine at position 360 in isoforms BCL11A-XL and -L, in a total of 26 individuals; two alleles at this multiallelic locus are classified as pathogenic, including the p.Leu360ProfsTer212 recurrent in our cohort. While sequencing artifacts are possible, two of the three patients in our cohort (all with a single nucleotide duplication, c.1078dup) had hemoglobin isoforms assessed, confirming persistence of HbF and supporting pathogenicity of the alleles inducing frameshift at the same locus. There are three alleles in gnomAD downstream of the variants identified in the BCL11A-IDD cohort and downstream of a putative TBR1 interaction domain of BCL11A-L (Fig. 1B) [37], and seven that spare the BCL11A-XL isoform, affecting BCL11A-L, with or without affecting BCL11A-S. HbF and neurocognitive assessment of these individuals would be useful to determine the impact of variants on BCL11A transcriptional activity, and provide further insight into the requirement of disruption of the putative TBR1 interaction domain for neurobehavioral and/or cognitive phenotypes. BCL11A’s intolerance to loss of function is underscored by the high pLI score of 1, and a LOEUF (loss-of-function observed / expected upper bound fraction) of 0.04. Given the variable severity of IDD and rareness of malformations we identify in BCL11A-IDD, we posit that individuals reported to have loss of function inducing variants in gnomAD had a mild neurodevelopmental phenotype not precluding their inclusion in gnomAD cohorts, and/or the variants have minimal phenotypic impact due to their C-terminal location or sparing of BCL11A-XL. Importantly, version v4 of gnomAD includes biobank samples, namely from the UK Biobank, which include disease samples without associated phenotypic metadata. Based on our current and previous evidence for loss of function and hypomorphic alleles disrupting BCL11A transcriptional activity [2], we suggest HbF levels be used as a proxy for loss of activity in clinical interpretation of putative loss of function *BCL11A* variants.

Our data and observations by Aldinger et al. [20] support the role of *BCL11A* in hindbrain abnormalities. While posterior fossa involvement (reduced vermis) is documented in the heterozygous mouse model [2], pons and medulla abnormalities have not been explored. Our findings may represent a mild pontocerebellar hypoplasia phenotype, which should be distinguished from classical forms of pontocerebellar hypoplasia (PCH) by absence of evolving cerebellar atrophy and associated supratentorial features [38]. In individuals with 2p16 deletions, haploinsufficiency of *BCL11A*, but not of adjacent coding or non-coding regions, is likely responsible for the rare cerebellar and pontine anomalies [20]. Interestingly, ataxia and/or spasticity were more frequent in previously reported patients. This may reflect a selection bias; four individuals listed in ClinVar as having IDD and spastic paraplegia were submitted by the same center (Supplementary Table 1). Nonetheless, neuroradiological findings in patients and BCL11A expression in human fetal cerebellar development are consistent with its importance in motor control. Longitudinal studies will be required to ascertain if patients who present with hypotonia, a common feature, develop motor impairment and spasticity with age.

On the other hand, cortical malformations are rare in BCL11A-IDD vs. large CNVs, suggesting that neocortex development is less sensitive to *BCL11A* haploinsufficiency with other genes in the 2p16p15 region playing a more significant role. Nevertheless, postnatal microcephaly emerged as a common and previously underappreciated feature of BCL11A-IDD. Despite its role in late differentiation and survival of upper layer neurons [4], and interaction with TBR1, a transcription factor critical for early-born neurons in the developing cortex in in vitro assays [37], heterozygous loss of *BCL11A* may not be sufficient to cause cortical malformations detectable by current imaging techniques.

Though birth defects are rare, polydactyly was identified in four independent individuals (Supplementary Table 1). *Bcl11a* is expressed in mouse developing limb buds, though limb anomalies have not been observed in mutant models [2, 39]. Further experimentation may be required to define the role of BCL11A in developing limbs and links to malformations. Increased frequency of malformations in patients with large CNVs encompassing additional coding genes suggests that other genes in the deleted region(s) may be responsible for congenital malformations. Our findings strengthen the proposition that BCL11A-IDD and larger deletions of 2p16.1p15 encompassing *BCL11A* with multiple adjacent genes should be considered two distinct conditions [1].

The frequency of strabismus and behavioral abnormalities/ASD warrant ophthalmologic and behavioral assessment at diagnosis, respectively, to ensure appropriate management. Features suggestive of autonomic dysfunction, reported in other neurodevelopmental syndromes [40, 41], are newly identified manifestations of BCL11A-IDD. In 1981 Manders et al. reported a child with IDD, persistent HbF and Raynaud’s phenomenon [42]; genetic testing of this patient has not been reported, though could represent the very first description of BCL11A-IDD or 2p16.1p15 deletion syndrome.

Elevated HbF, reported in *BCL11A* variants and microdeletions [2, 8, 43], is also observed in hereditary persistence of fetal hemoglobin (HPFH) and *ZBTB7A*-related neurodevelopmental disorder [OMIM#619769]. BCL11A-IDD is distinguished from the latter as *ZBTB7A*-NDD is characterized by macrocephaly and hypertrophy of pharyngeal lymphoid tissue [44]. HPHF is a benign condition usually caused by deletions encompassing the β-globin gene cluster or by SNVs in the γ-globin gene-promoter region. Although HPFH and BCL11A-IDD share the elevated HbF trait, HPFH lacks the cognitive and systemic features discussed above. As previously identified in individuals with HPFH in association with sickle cell anemia, we identified a negative correlation between HbF levels and age [45], though its significance in the absence of concomitant hematological disease is unknown. We suggest using HbF testing by hemoglobin electrophoresis or HPLC as a biomarker for BCL11A-IDD with variants of unknown significance, though physiologically elevated HbF in the general population in the first 12–24 months of life precludes it as an early infant biomarker. It is possible that BCL11A-IDD could mask HPFH in individuals where persistent HbF has dual etiology, hence, parental HbF testing may be offered where relevant.

In conclusion, our study provides a comprehensive understanding of the BCL11A-IDD phenotype in the largest cohort reported to date. Our recognition of previously underappreciated manifestations, such as postnatal microcephaly, behavior abnormalities, seizures, abnormal motor function, and autonomic dysregulation, expands the phenotypic spectrum. Brain imaging analysis underscored cerebellar and posterior fossa abnormalities as a prevalent manifestation of BCL11A-IDD, supporting the role of *BCL11A* in hindbrain development. We expand the *BCL11A* mutational spectrum and find that protein-truncating variants tend to be associated with a more severe phenotype compared to the other variant types and that BCL11A-S retention may contribute to protein dysfunction. Longitudinal assessments of our cohort through adulthood or identification of larger numbers of affected adults will be required to determine additional features, such as progressive neurologic manifestations or scoliosis, and to exclude the possibility of an increased risk of malignancy and immunologic disease. To further our understanding of BCL11A-IDD, we founded the publicly available website https://humandiseasegenes.nl/bcl11a collecting genotypic and phenotypic data. Additional investigation into the pathogenesis of disease in human in vitro models may permit further characterization of the molecular and cellular defects produced by *BCL11A* haploinsufficiency in human brain development.

### Online resources referenced

www.ensembl.org/

https://humandiseasegenes.nl/bcl11a

https://deciphergenomics.org/

https://omim.org/entry/617101

https://gnomad.broadinstitute.org/gene/ENSG00000119866?dataset=gnomad_r4

https://db.gestaltmatcher.org/

## Supplementary information

Supplementary Material

Supplementary Material

## Data Availability

All clinical and molecular data included in this study are provided in Supplementary Material and Supplementary Tables. Aggregate data is publicly available at https://humandiseasegenes.nl/bcl11a. Genetic analyses had been previously performed. For access, please contact the authors who will refer to the appropriate center (data availability is subject to each individual study’s ethics and consent).

## References

[1] Peron A, Bradbury K, Viskochil DH, Dias C. BCL11A-Related Intellectual Disability. In: Adam MP, Ardinger HH, Pagon RA, Wallace SE, Bean LJH, Mirzaa G, et al., editors. GeneReviews(®). Seattle (WA): University of Washington, Seattle; 2019. 31556984

[2] Dias C, Estruch SB, Graham SA, McRae J, Sawiak SJ, Hurst JA, et al. BCL11A Haploinsufficiency Causes an Intellectual Disability Syndrome and Dysregulates Transcription. Am J Hum Genet. 2016;99:253–74. 27453576 10.1016/j.ajhg.2016.05.030 PMC4974071

[3] Liu N, Hargreaves VV, Zhu Q, Kurland JV, Hong J, Kim W, et al. Direct Promoter Repression by BCL11A Controls the Fetal to Adult Hemoglobin Switch. Cell. 2018;173:430–42.e17. 29606353 10.1016/j.cell.2018.03.016 PMC5889339

[4] Wiegreffe C, Simon R, Peschkes K, Kling C, Strehle M, Cheng J, et al. Bcl11a (Ctip1) Controls Migration of Cortical Projection Neurons through Regulation of <em>Sema3c</em>. Neuron 2015;87:311–25. 26182416 10.1016/j.neuron.2015.06.023

[5] Woodworth MB, Greig LC, Liu KX, Ippolito GC, Tucker HO, Macklis JD. Ctip1 Regulates the Balance between Specification of Distinct Projection Neuron Subtypes in Deep Cortical Layers. Cell Rep. 2016;15:999–1012. 27117402 10.1016/j.celrep.2016.03.064 PMC4873759

[6] De Rubeis S, He X, Goldberg AP, Poultney CS, Samocha K, Ercument Cicek A, et al. Synaptic, transcriptional and chromatin genes disrupted in autism. Nature. 2014;515:209–15. 25363760 10.1038/nature13772 PMC4402723

[7] The Deciphering Developmental Disorders S. Large-scale discovery of novel genetic causes of developmental disorders. Nature. 2015;519:223–8. 25533962 10.1038/nature14135 PMC5955210

[8] Basak A, Hancarova M, Ulirsch JC, Balci TB, Trkova M, Pelisek M, et al. BCL11A deletions result in fetal hemoglobin persistence and neurodevelopmental alterations. J Clin Investig. 2015;125:2363–8. 25938782 10.1172/JCI81163 PMC4497765

[9] Soblet J, Dimov I, Graf von Kalckreuth C, Cano-Chervel J, Baijot S, Pelc K, et al. BCL11A frameshift mutation associated with dyspraxia and hypotonia affecting the fine, gross, oral, and speech motor systems. Am J Med Genet Part A. 2018;176:201–8. 28960836 10.1002/ajmg.a.38479 PMC5765401

[10] Wessels MW, Cnossen MH, van Dijk TB, Gillemans N, Schmidt KLJ, van Lom K, et al. Molecular analysis of the erythroid phenotype of a patient with BCL11A haploinsufficiency. Blood Adv. 2021;5:2339–49. 33938942 10.1182/bloodadvances.2020003753 PMC8114548

[11] Yoshida M, Nakashima M, Okanishi T, Kanai S, Fujimoto A, Itomi K, et al. Identification of novel BCL11A variants in patients with epileptic encephalopathy: Expanding the phenotypic spectrum. Clin Genet. 2018;93:368–73. 28589569 10.1111/cge.13067

[12] Korenke GC, Schulte B, Biskup S, Neidhardt J, Owczarek-Lipska M. A Novel de novo Frameshift Mutation in the BCL11A Gene in a Patient with Intellectual Disability Syndrome and Epilepsy. Mol Syndromol. 2020;11:135–40. 32903878 10.1159/000508566 PMC7445578

[13] Cai T, Chen X, Li J, Xiang B, Yang L, Liu Y, et al. Identification of novel mutations in the HbF repressor gene BCL11A in patients with autism and intelligence disabilities. Am J Hematol. 2017;92:E653–E6. 28891213 10.1002/ajh.24902

[14] Shen Y, Li R, Teichert K, Montbleau KE, Verboon JM, Voit RA, et al. Pathogenic BCL11A variants provide insights into the mechanisms of human fetal hemoglobin silencing. PLOS Genet. 2021;17:e1009835. 34634037 10.1371/journal.pgen.1009835 PMC8530301

[15] Satterwhite E, Sonoki T, Willis TG, Harder L, Nowak R, Arriola EL, et al. The BCL11 gene family: involvement of BCL11A in lymphoid malignancies. Blood. 2001;98:3413–20. 11719382 10.1182/blood.v98.12.3413

[16] Sobreira N, Schiettecatte F, Valle D, Hamosh A. GeneMatcher: A Matching Tool for Connecting Investigators with an Interest in the Same Gene. Hum Mutat. 2015;36:928–30. 26220891 10.1002/humu.22844 PMC4833888

[17] Swaminathan GJ, Bragin E, Chatzimichali EA, Corpas M, Bevan AP, Wright CF, et al. DECIPHER: web-based, community resource for clinical interpretation of rare variants in developmental disorders. Hum Mol Genet. 2012;21:R37–44. 22962312 10.1093/hmg/dds362 PMC3459644

[18] Landrum MJ, Lee JM, Benson M, Brown G, Chao C, Chitipiralla S, et al. ClinVar: public archive of interpretations of clinically relevant variants. Nucleic Acids Res. 2016;44:D862–8. 26582918 10.1093/nar/gkv1222 PMC4702865

[19] Beleford DT, Van Ziffle J, Hodoglugil U, Slavotinek AM. A missense variant, p.(Ile269Asn), in MC4R as a secondary finding in a child with BCL11A-related intellectual disability. Eur J Med Genet. 2020;63:103969. 32534219 10.1016/j.ejmg.2020.103969

[20] Aldinger KA, Timms AE, Thomson Z, Mirzaa GM, Bennett JT, Rosenberg AB, et al. Redefining the Etiologic Landscape of Cerebellar Malformations. Am J Hum Genet. 2019;105:606–15. 31474318 10.1016/j.ajhg.2019.07.019 PMC6731369

[21] Ottolini KM, Turner CE, Gada SM. Hypogammaglobulinemia and impaired antibody response in a child with chromosome 2p15-16.1 microdeletion syndrome. Ann Allergy Asthma Immunol. 2015;115:153–5. 26100565 10.1016/j.anai.2015.05.016

[22] Carey JC, Allanson JE, Hennekam RCM, Biesecker LG. Standard terminology for phenotypic variations: The Elements of Morphology project, its current progress, and future directions. Hum Mutat. 2012;33:781–6. 22331827 10.1002/humu.22053

[23] Köhler S, Gargano M, Matentzoglu N, Carmody LC, Lewis-Smith D, Vasilevsky NA, et al. The Human Phenotype Ontology in 2021. Nucleic Acids Res. 2020;49:D1207–D17. 10.1093/nar/gkaa1043 PMC7778952 33264411

[24] Vogel M. Childsds: data and methods around reference values in pediatrics. 2020. https://cran.r-project.org/web/packages/childsds/index.html.

[25] Lindeboom RGH, Supek F, Lehner B. The rules and impact of nonsense-mediated mRNA decay in human cancers. Nat Genet. 2016;48:1112–8. 27618451 10.1038/ng.3664 PMC5045715

[26] Jandeaux C, Kuchcinski G, Ternynck C, Riquet A, Leclerc X, Pruvo J-P, et al. Biometry of the Cerebellar Vermis and Brain Stem in Children: MR Imaging Reference Data from Measurements in 718 Children. Am J Neuroradiol. 2019;40:1835–41. 31624120 10.3174/ajnr.A6257 PMC6975126

[27] Severino M, Huisman TAGM. Posterior Fossa Malformations. Neuroimaging Clin North Am. 2019;29:367–83. 10.1016/j.nic.2019.03.008 31256860

[28] Cheng J, Novati G, Pan J, Bycroft C, Žemgulytė A, Applebaum T, et al. Accurate proteome-wide missense variant effect prediction with AlphaMissense. Science. 2023;381:eadg7492. 37733863 10.1126/science.adg7492

[29] Yang Y, Xu Z, He C, Zhang B, Shi Y, Li F. Structural insights into the recognition of γ-globin gene promoter by BCL11A. Cell Res. 2019;29:960–3. 31467406 10.1038/s41422-019-0221-0 PMC6888818

[30] Aldinger KA, Thomson Z, Phelps IG, Haldipur P, Deng M, Timms AE, et al. Spatial and cell type transcriptional landscape of human cerebellar development. Nat Neurosci. 2021;24:1163–75. 34140698 10.1038/s41593-021-00872-y PMC8338761

[31] Haldipur P, Aldinger KA, Bernardo S, Deng M, Timms AE, Overman LM, et al. Spatiotemporal expansion of primary progenitor zones in the developing human cerebellum. Science. 2019;366:454–60. 31624095 10.1126/science.aax7526 PMC6897295

[32] Hsieh T-C, Bar-Haim A, Moosa S, Ehmke N, Gripp KW, Pantel JT, et al. GestaltMatcher facilitates rare disease matching using facial phenotype descriptors. Nat Genet. 2022;54:349–57. 35145301 10.1038/s41588-021-01010-x PMC9272356

[33] Hustinx A, Hellmann F, Sumer O, Javanmardi B, Andre E, Krawitz P, et al. Improving deep facial phenotyping for ultrarare disorder verification using model Ensembles. In: 2023 IEEE/CVF winter conference on applications of computer vision (WACV). IEEE; 2023.

[34] Kingdom R, Tuke M, Wood A, Beaumont RN, Frayling TM, Weedon MN, et al. Rare genetic variants in genes and loci linked to dominant monogenic developmental disorders cause milder related phenotypes in the general population. Am J Hum Genet. 2022;109:1308–16. 35700724 10.1016/j.ajhg.2022.05.011 PMC9300873

[35] Teran NA, Nachun DC, Eulalio T, Ferraro NM, Smail C, Rivas MA, et al. Nonsense-mediated decay is highly stable across individuals and tissues. Am J Hum Genet 2021;108:1401–08. 34216550 10.1016/j.ajhg.2021.06.008 PMC8387471

[36] Karczewski KJ, Francioli LC, Tiao G, Cummings BB, Alföldi J, Wang Q, et al. The mutational constraint spectrum quantified from variation in 141,456 humans. Nature. 2020;581:434–43. 32461654 10.1038/s41586-020-2308-7 PMC7334197

[37] den Hoed J, Sollis E, Venselaar H, Estruch SB, Deriziotis P, Fisher SE. Functional characterization of TBR1 variants in neurodevelopmental disorder. Sci Rep. 2018;8:14279. 30250039 10.1038/s41598-018-32053-6 PMC6155134

[38] Rudnik-Schöneborn S, Barth PG, Zerres K. Pontocerebellar hypoplasia. Am J Med Genet Part C Semin Med Genet. 2014;166:173–83. 10.1002/ajmg.c.31403 24924738

[39] Liu P, Keller JR, Ortiz M, Tessarollo L, Rachel RA, Nakamura T, et al. Bcl11a is essential for normal lymphoid development. Nat Immunol. 2003;4:525–32. 12717432 10.1038/ni925

[40] Weese-Mayer DE, Lieske SP, Boothby CM, Kenny AS, Bennett HL, Ramirez J-M. Autonomic dysregulation in young girls with Rett Syndrome during nighttime in-home recordings. Pediatr Pulmonol. 2008;43:1045–60. 18831533 10.1002/ppul.20866

[41] Zollino M, Zweier C, Van Balkom ID, Sweetser DA, Alaimo J, Bijlsma EK, et al. Diagnosis and management in Pitt-Hopkins syndrome: First international consensus statement. Clin Genet. 2019;95:462–78. 30677142 10.1111/cge.13506

[42] Manders AJ, von Oostrom CG, Trijbels JMF, Rutten FJ, Kleijer WJ. α-aminoadipic aciduria and persistence of fetal haemoglobin in an oligophrenic child. Eur J Pediatrics. 1981;136:51–5. 10.1007/BF00441711 6163632

[43] Funnell APW, Prontera P, Ottaviani V, Piccione M, Giambona A, Maggio A, et al. 2p15-p16.1 microdeletions encompassing and proximal to BCL11A are associated with elevated HbF in addition to neurologic impairment. Blood. 2015;126:89–93. 26019277 10.1182/blood-2015-04-638528 PMC4492199

[44] von der Lippe C, Tveten K, Prescott TE, Holla ØL, Busk ØL, Burke KB, et al. Heterozygous variants in ZBTB7A cause a neurodevelopmental disorder associated with symptomatic overgrowth of pharyngeal lymphoid tissue, macrocephaly, and elevated fetal hemoglobin. Am J Med Genet Part A. 2022;188:272–82. 34515416 10.1002/ajmg.a.62492

[45] Ngo DA, Aygun B, Akinsheye I, Hankins JS, Bhan I, Luo HY, et al. Fetal haemoglobin levels and haematological characteristics of compound heterozygotes for haemoglobin S and deletional hereditary persistence of fetal haemoglobin. Br J Haematol. 2012;156:259–64. 22017641 10.1111/j.1365-2141.2011.08916.x PMC6605093

