# Supplementary material for: BCL11A intellectual developmental disorder: defining the clinical spectrum and genotype-phenotype correlations
Source: Eur J Hum Genet. 2024 Oct 24;33(3):312–24. doi: 10.1038/s41431-024-01701-z (PMC11893779; doi:10.1038/s41431-024-01701-z)
Supplement: Supplementary file 1 — Supplementary Material [file 41431_2024_1701_MOESM1_ESM.docx]

## Supplementary Material for

**BCL11A intellectual developmental disorder: defining the clinical spectrum and genotype-phenotype correlations**

Peron A. et al, 2024

**A. Supplementary Figures**

Supplementary Figure 1: BCL11A-IDD copy number variants and analysis of selected variants; supplementary figure to Figure 1.

Supplementary Figure 2: Physical features of individuals with BCL11A-IDD in the combined dataset.

Supplementary Figure 3: GestaltMatcher mean pairwise distance and mutation type analyses.

Supplementary Figure 4: GestaltMatcher hierarchical clustering analysis

**B. Supplementary Tables**

Supplementary Table 1: Clinical features of the present cohort and previously reported individuals - *provided as separate excel file*

Supplementary Table 2: Protein truncating variant (PTV) categories and predicted effect on encoded protein.

Supplementary Table 3: Sequence variant annotations for BCL11A-XL, -L and -S isoforms - *provided as separate excel file*

Supplementary Table 4: Copy number variants in *BCL11A* - *provided as separate excel file*

Supplementary Table 5: Summary of brain MRI quantitative and qualitative analysis findings.

Supplementary Table 6: Summary of congenital malformations and brain MRI findings in previously reported individuals with large deletions encompassing additional genes - *provided as separate excel file*

Supplementary Table 7: Sequential HbF measurements

Supplementary Table 8: Loss of function sequence variants in *BCL11A* in GnomAD(1) - *provided as separate excel file*

Supplementary Table 9: Immunohistochemistry antibodies used.

**C. Supplementary Materials and Methods.**

**D. Supplementary Information – consortia members.**

**E. Supplementary Reference List.**

### A. Supplementary Figures


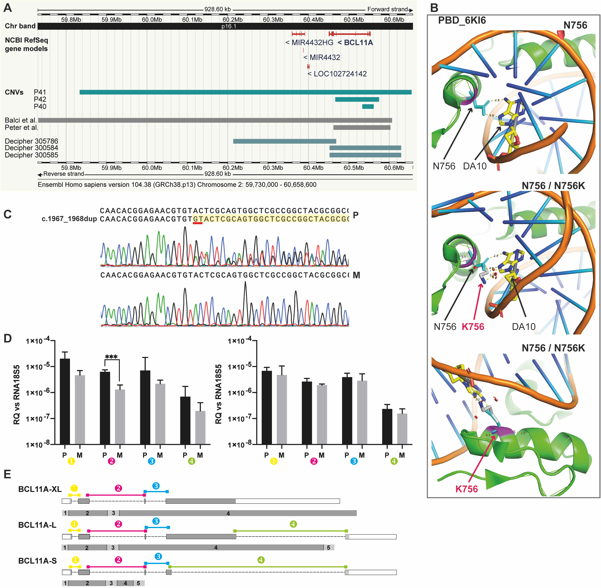


*Supplementary Figure 1.* BCL11A-IDD copy number variants and analysis of selected variants; supplementary figure to Figure 1.

**A.** CNVs presented in this manuscript. Figure adapted from www.ensembl.org/. **B.** Asn756Lys mutation modelled on PBD 6KI6 (Crystal structure of BCL11A in complex with gamma-globin -115 HPFH region***(2)***). The N756 residue (magenta and cyan) is located in the fourth C2H2 zinc finger domain (green) of BCL11A-XL (see Figure 1) which binds the proximal promoter of the γ-globin gene cluster (DNA helix in gold and blue). Substitution by a large lysine (magenta and white; middle and right panels) is sterically unfavorable, altering the interaction with the N6 and N7 atoms of adenine D10 (yellow and blue, middle) and with adjacent ZNF amino acids (right). **C.** dideoxynucleotide sequencing of patient cDNA (P, top) showing a heterozygous GT duplication (underlined in red) causing a frameshift (yellow) in comparison with reference sequence above; bottom, maternal cDNA (M). **D.** rt-PCR (mean ±SD of 3 technical replicates) of lymphoblastoid cell lines in patient 32 (P) harboring a heterozygous variant (*BCL11A*:c.1967_1968dup, p.Ser657ThrfsTer134) and unaffected mother (M) at one (left) and eight (right) passages post-seeding; numbers indicate probes used. **E.** Diagram representing three major BCL11A isoforms (greys, coding exons) and exon-spanning TaqMan® probes (1, Hs00256254_m1; 2, Hs01093196_m1; 3, Hs01093197_m1; 4, Hs01093199_m1; Supplementary Methods).


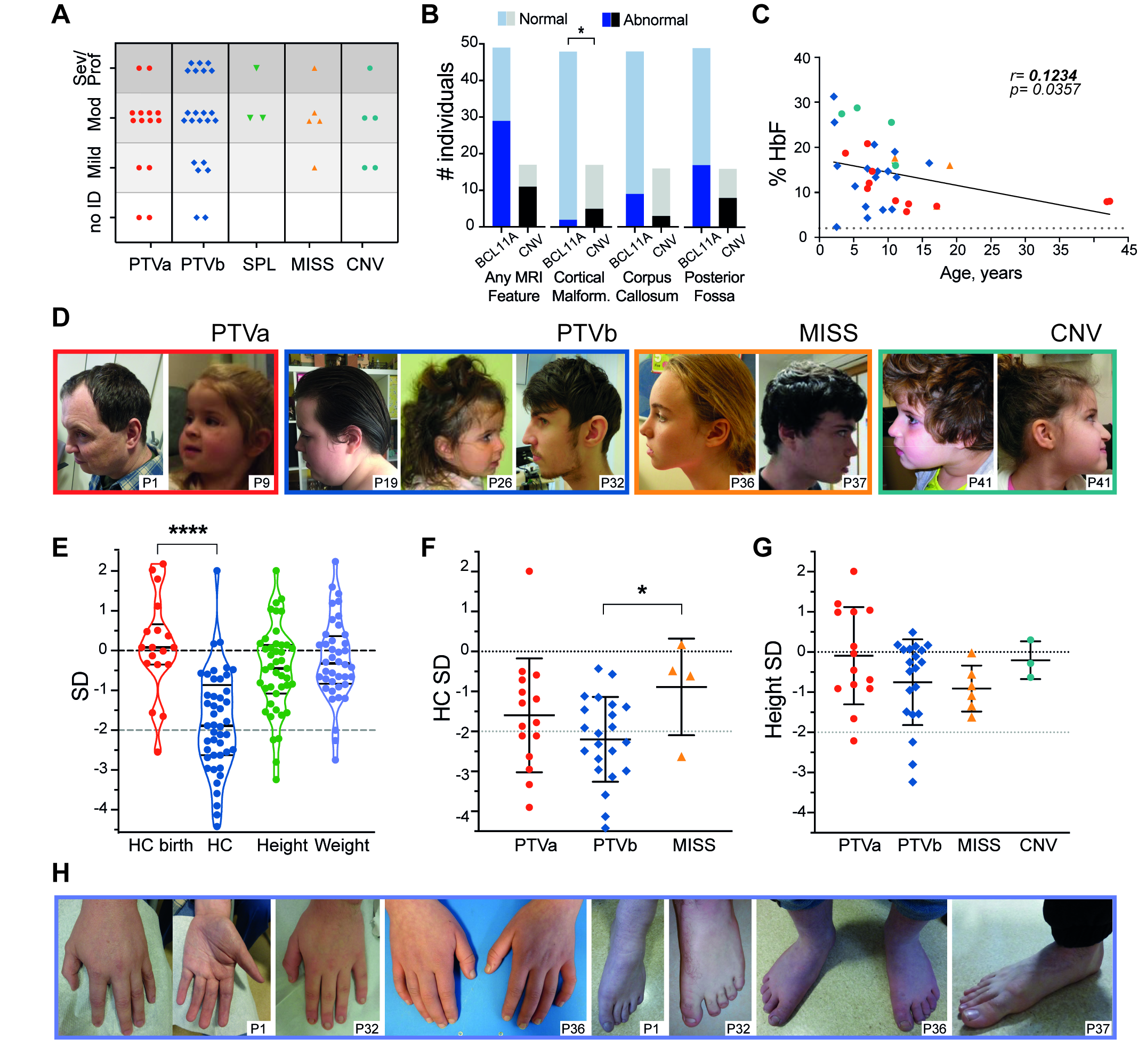


***Supplementary Figure 2.*** **Physical features of individuals with BCL11A-IDD in the combined dataset.**

**A.** Severity of intellectual disability per mutation type for all individuals in whom severity was available; no significant difference between severity and mutation type (Mann Whitney tests not significant for p<0.05). **B.** Brain MRI abnormalities in individuals with *BCL11A* mutations and small deletions and in individuals with large CNVs encompassing *BCL11A* and contiguous genes (Supplementary Table 4). **p=* *0.0109* (Fisher’s exact test). **C.** Fetal hemoglobin (HbF) for age; all available data is presented (present and previously reported cohort; multiple measurements per individual where available); dotted line: reference cutoff of 2%; black line: linear regression, *r^2^=0.1234*; *p=0.0357*; Spearman correlation coefficient *r=-0.3274, p=0.0513*. Symbols: red circles, PTVa; blue lozenges, PTVb; yellow triangles, MISS; cyan circles, CNV; inverted green triangle, SPL. **D.** Craniofacial features (profile) of selected individuals with *BCL11A* variants in the present cohort; patient number on bottom right; mutation class indicated by colored outline. Approximate ages at time of photographs (y, years; m, months): P1, 42y; P9, 3y2m; P19, 16y; P26, 4y6m; P32, 18y; P36, 11y2m; P37, 16y; P41 3y3m (left), 7y8m (right) **E.** Distribution of growth parameters of present cohort and previously reported cases (a single most recent value for each individual is represented); lines indicate median, upper and lower quartiles; ****unpaired t-test *p<0.0001*. **F.** Head circumference SDs for PTV classes a and b and MISS; n=15 PTVa, 22 PTVb, 4 MISS; unpaired t test: **p=0.0353*. **G.** Height SD per mutation type; n=14 PTVa, 19 PTVb, 6 MISS, 3 CNV; 1 SPL not represented (data in Supplementary Table 1). **H.** Hands and feet of selected individuals with *BCL11A* variants in the present cohort.

**
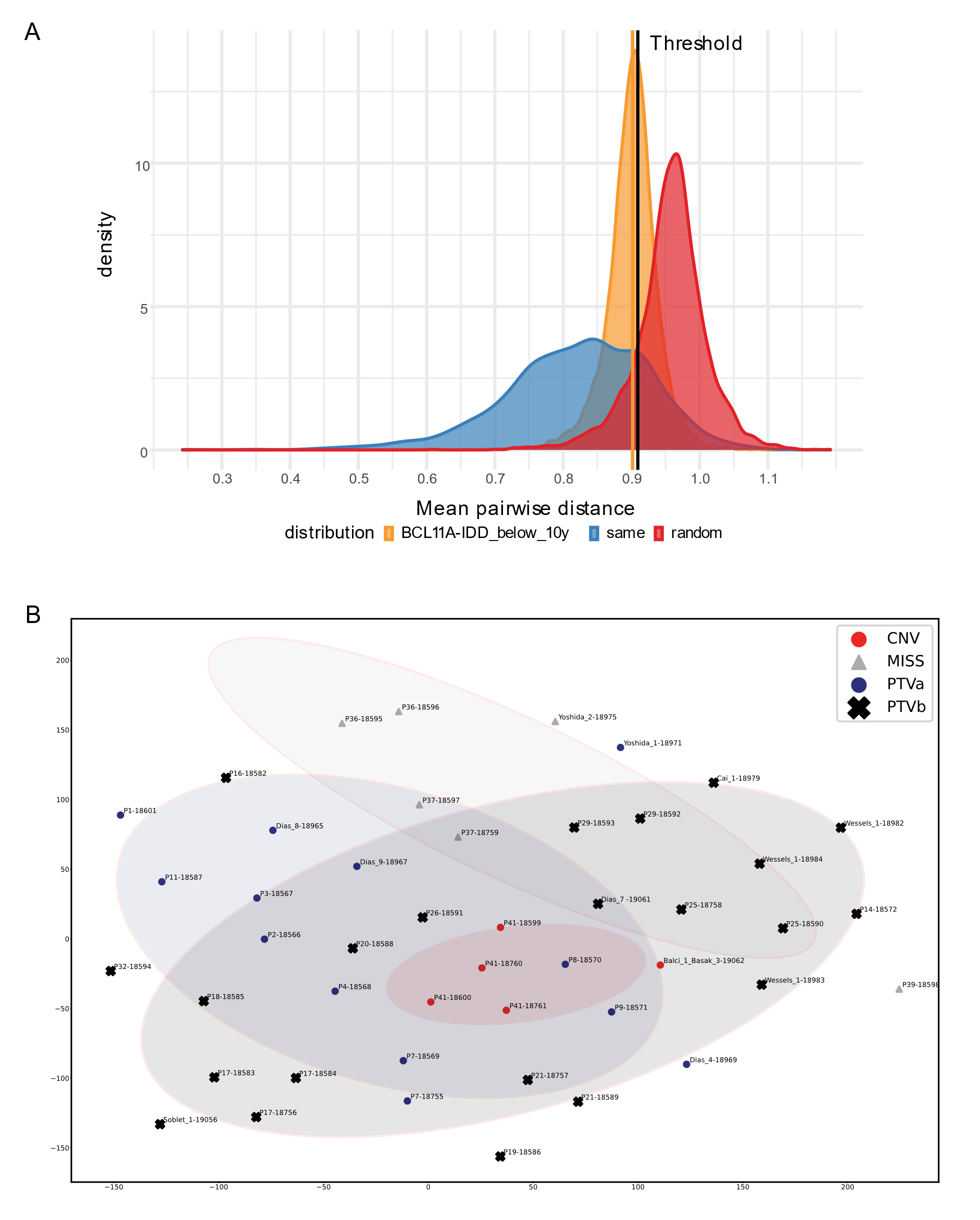
**

***Supplementary Figure 3.* GestaltMatcher mean pairwise distance and mutation type analyses.**

**A.** The distribution of mean pairwise distance. It shows three distributions: BCL11A-IDD patients below ten years old (orange), the random selection from the subjects with 328 disorders (red), and the selection with the same disorder (blue). The black vertical line is the threshold that classifies whether it is the same disorder or random selection. 58.8% of BCL11A-IDD distribution are below the threshold; **B.** tSNE plot of BCL11A-IDD with different types of mutations. Each dot is an image, and the label consists of an individual ID and the GestaltMatcher Database (GMDB) image ID that can be used to check the image in GMDB.


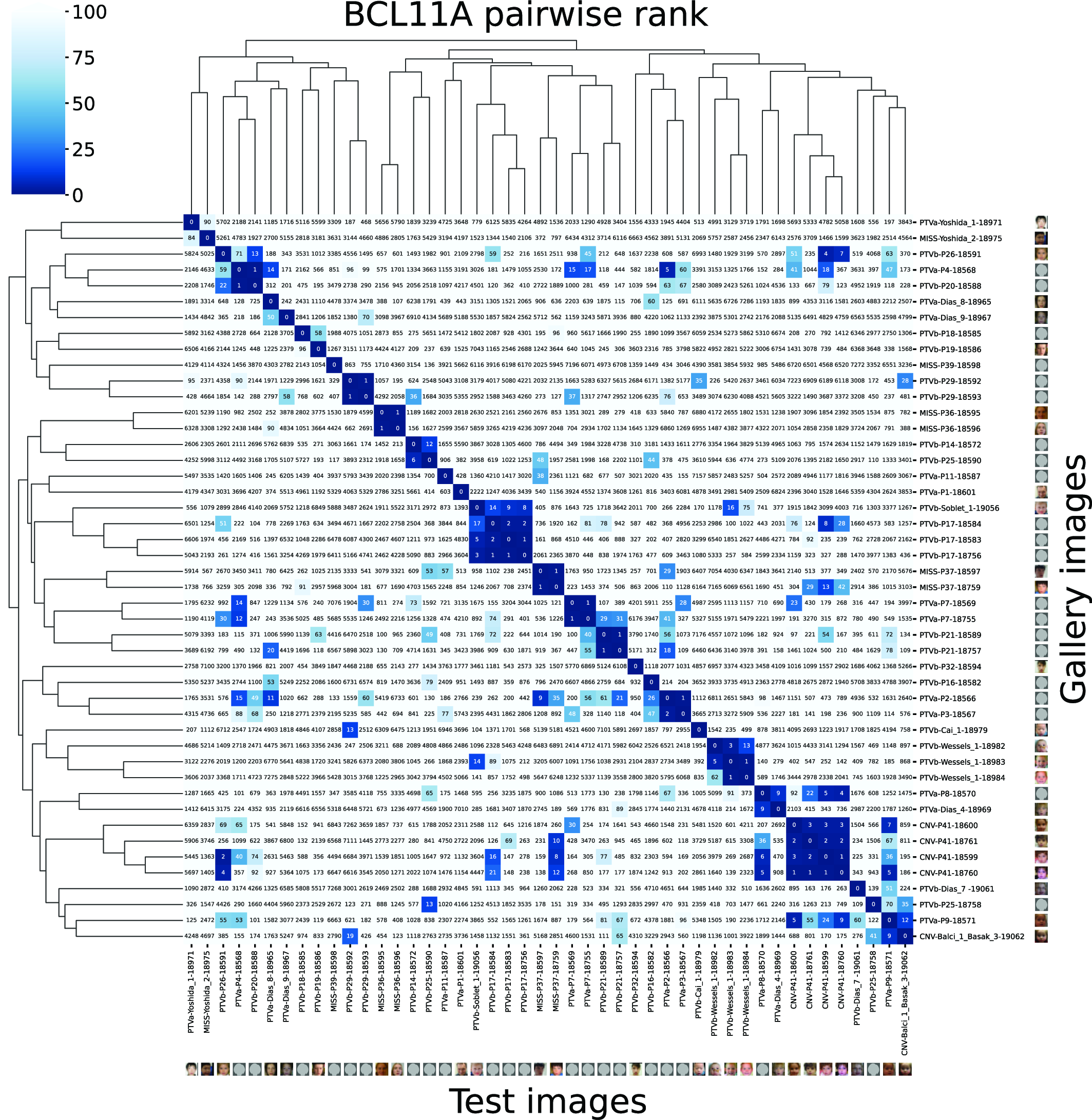


***Supplementary Figure 4.* GestaltMatcher hierarchical clustering analysis.**

The pairwise rank matrix and hierarchical clustering of 46 images of 33 individuals with BCL11A-IDD. Each column is the result of testing one image in the column and listing the rank of the rest of the 45 images in each row. The labeling consists of mutation type, individual ID, and GMDB ID. GMDB ID can be used to check the image in GMDB and distinguish the multiple images from the same individual. For example, by testing Ind P9, Individual Balci_1_Basak_3 was on the 9^th^ rank, and P41 with image ID 18760 was on the 5^th^ rank of Ind P9.

### B. Supplementary Tables

|  |  | ***BCL11A-S*** | ***BCL11A-L*** | ***BCL11A-XL*** | **n-ZNF^1^** | **c-ZNF^2^** |
| --- | --- | --- | --- | --- | --- | --- |
| **PTVa** |  | **PTC** | **PTC** | **PTC** | upstream | upstream |
|  | **PTVa1** | NMDe SP/LE | NMDe SP/LE | NMDe SP/LE |  |  |
|  | **PTVa2** | NMD**+** | NMD/NMDe | NMD/NMDe |  |  |
| **PTVb** |  | **unaffected** | **PTC** | **PTC** |  |  |
|  | **PTVb1** |  | NMDe EL | NMDe LE | upstream | upstream |
|  | **PTVb2** |  | NMDe EL/LE/EJ | NMDe LE | downstream | upstream |

#### ****Supplementary Table**** 2. PTV categories and predicted effect on encoded protein.

*BCL11A-S* (NM_138559.2) encodes BCL11A-S (NP_612569.1, 243 aa). *BCL11A-L* (NM_018014.4) encodes BCL11A-L (NP_060484.2, 773 aa). BCL11A-XL (NM_022893.4, MANE transcript) encodes BCL11A-XL (NP_075044.2, 835 aa). See figure 1. ^1^N-terminus ZNF cluster. ^2^C-terminus ZNF cluster. PTC: premature termination codon. NMD**+**: nonsense mediated decay. NMDe: expected to escape NMD (significantly truncated protein): SP, start proximal; LE, last exon; EL, long exon (exon length ≥407 nt); EJ, exon juntion (≤ 50 nt from last exon junction).

| **Patient ID** | **Mutation, NP_075044.2:** | **Mutation class** | **Age** | **Posterior fossa findings** | **Supratentorial findings** |
| --- | --- | --- | --- | --- | --- |
| **P3** | p.Gly7AlafsTer9 | PTVa1 | 6y | small pons relative to medulla and in comparison with normal controls, small vermis height, mildly hypoplastic left cerebellar hemisphere | normal |
| **P6** | p.Met53ProfsTer32 | PTVa2 | 10m | vermian hypoplasia and small pons | small corpus callosum |
| **P8** | p.Val99TrpfsTer29 | PTVa2 | 1y 6m | small pons relative to medulla and in comparison with normal controls, small vermis height | normal |
| **P12** | p.Gly212ArgfsTer21 | PTVb1 | 10y | small pons relative to medulla and in comparison with normal controls, small vermis height | dysmorphic corpus callosum |
| **P17** | p.Leu360ProfsTer212 | PTVb1 | 3y 5m | small pons relative to medulla and in comparison with normal controls, small vermis height | dysmorphic corpus callosum, incomplete hippocampal rotation bilaterally |
| **P20** | p.Val374GlyfsTer198 | PTVb1 | NA^1^ | normal | normal |
| **P25** | p.Val534AlafsTer54 | PTVb2 | NA^1^ | small pons relative to medulla and in comparison with normal controls, small vermis height | normal |
| **P30** | p.Ser679GlnfsTer47 | PTVb2 | 8m | normal | minor white matter abnormalities |
| **P35** | c.385+2T>C | SPL | 2y | small pons relative to medulla and in comparison with normal controls, small vermis height, hypoplastic left cerebellar hemisphere, abnormal vermis foliation | dysmorphic corpus callosum |
| **P36** | p.Thr47Pro | MISS | 1y 8m | normal | slightly reduced white matter volume |
| **P37** | p.Cys48Phe | MISS | 3y | small pons relative to medulla and in comparison with normal controls, small vermis height | normal |
| **P40** | partial gene deletion | CNV | 1y  10m | slightly small pons on measurements | left fronto insular closed lip schizencephaly |
| **P41** | whole gene deletion | CNV | 2y | small pons relative to medulla and in comparison with normal controls, small vermis height, retrocerebellar cyst | dysmorphic corpus callosum |
| **Dias_4** | p.Gln177Ter | PTVa1 | 2y | slightly small pons on measurements | normal |
| **Balci_1** | whole gene deletion | CNV | 1y 3m | small pons relative to medulla and in comparison with normal controls, small vermis height and Blake’s pouch cyst (BPC) | hydrocephalus likely associated to the BPC, bilateral malrotation of the hippocampi |

#### ****Supplementary Table**** 5.

Summary of brain MRI quantitative and qualitative analysis findings.

^1^Age at scan and full DICOM file not available; selected images analyzed only.

| **Patient ID** | **Sex** | **Age (yrs)** | **HbF** |
| --- | --- | --- | --- |
| **P1** | M | 41.9 | 8% |
|  |  | 42.3 | 8.1% |
| **P17** | M | 2.2 | 25.5% |
|  |  | 2.6 | 15.9% |
|  |  | 5.2 | 11.4% |
|  |  | 6.75 | 6.9% |
|  |  | 9.25 | 6.2% |
|  |  | 10.6 | 6.3% |
| **P21** | F | 2.1 | 31.2% |
|  |  | 8 | 20.6% |
|  |  | 8.2 | 13.4% |
|  |  | 8.5 | 14.7% |
|  |  | 11.25 | 13.4% |
| **P37** | M | 11.1 | 8.2% |
|  |  | 16.0 | 6.6% |
|  |  | 17.1 | 7% |
| **P41** | F | 3.25 | 27.4% |
|  |  | 5.5 | 28.7% |
|  |  | 10.5 | 25.5% |
| **Wessels_1** | F | 3.8 | 18.7% |
|  |  | 7.25 | 12.1% |

***Supplementary Table 7.*** Sequential HbF measurements for a subset of individuals where data was available. Normal reference value <2%.

| **Target** | **Host Species** | **Conjugate** | **Dilution** | **Company, catalogue number (clone ID), [RRID]** | **Tissue** |
| --- | --- | --- | --- | --- | --- |
| BCL11A (Ctip1) | mouse | NA | 1:50 | Abcam, ab19487 (14B5), [AB_444947] | a), d) |
| BCL11A (Ctip1) | rabbit | NA | 1:100 | Abcam, ab19489 (18B12DE6), [AB_2063996] | b), c) |
| Calbindin-D-28-K | rabbit | NA | 1:100 | Sigma-Aldrich, C9848 (CB-955), [AB_476894 | d) |
| Calbindin D-28k | rabbit | NA | 1:3000 | Swant, CD38, [AB_10000340] | b), c) |
| Ki67 | mouse | NA | 1:50 | DAKO, M7240 (MIB1), [AB_2142367] | b), c) |
| Ki67 | rat | NA | 1:50 | Thermo Fisher Scientific, 14-5698-82 (SolA15), [AB_10854564] | a), d) |
| NeuN | rabbit | NA | 1:50 | Abcam, ab177487 (EPR12763), [AB_2532109] | a), d) |
| SOX2 | rat | NA | 1:50 | Thermo Fisher Scientific, 14-9811-82 (Btjce), [AB_11219471] | a), d) |
| Tbr1 | rabbit | NA | 1:50 | Abcam, ab31940, [AB_2200219] | a) |
| βIII Tubulin | mouse | NA | 1:1000 | Promega, G712A (5G8), [AB_430874] | b), c) |
| Mouse IgG | donkey | AF 488 | 1:500 | Molecular Probes, A21202, [AB_141607] |  |
| Mouse IgG | goat | AF 568 | 1:500 | Molecular Probes, A11004, [AB_2534072] |  |
| Rabbit IgG | donkey | AF 546 | 1:500 | Thermo Fisher Scientific, A10040, [AB_2534016] |  |
| Rabbit IgG | goat | AF 488 | 1:500 | Thermo Fisher Scientific, A11034, [AB_2576217] |  |
| Rat IgG | donkey | AF 549 | 1:500 | Jackson ImmunoResearch Labs, 712-585-153, [AB_2340689] |  |

#### Supplementary Table 9.

Immunohistochemistry antibodies used. NA: not applicable (unconjugated). AF: Alexa Fluor. a) cortex, 15pcw; b) cerebellum 12pcw; c) cerebellum 16pcw; d) cerebellum 18pcw.

### C. Supplementary Materials and Methods

***In silico analysis*** of mutation *BCL11A* c.2268T>C, p.Asn756Lys (P39) was performed using The PyMOL Molecular Graphics System Version 2.4.1. Reference structure PDB 6KI6 was used (PDB doi:10.2210/pdb6KI6/pdb; crystal structure of BCL11A in complex with gamma-globin -115 HPFH region).(2)

***Lymphoblastoid cell lines***

Lymphoblastoid cell lines (LCL) for patient P32 and unaffected parent were obtained from human blood by immortalization via EBV-transformation according to standard procedures. Cells were grown in suspension in RPMI 1640 medium (Thermo Fisher Scientific Inc., Waltham, MA, USA; 11875093) supplemented with 15% FCS (Life Technologies, Life Technologies; 10270), 10mM HEPES (Gibco; 15630) and 100 U/mL Penicillin-Streptomycin (Gibco; Thermo Fisher Scientific; 15140122) at 37°C and 5% CO_2_.

***Reverse transcription PCR and cDNA sequencing***

Total cell RNA was extracted according to manufacturer’s instructions a) manually, using the Qiagen RNeasy Plus mini kit with gDNA Eliminator columns (Qiagen, Aarhus, Denmark; 74034), or b) on the QIAcube (Qiagen) using the Qiagen RNeasy Plus mini with on-column DNase I treatment. Complementary DNA (cDNA) was synthesized using Maxima First Strand cDNA Synthesis Kit (Thermo Fisher Scientific; K1672). RT-qPCR was undertaken using gene-specific TaqMan® Assay probes (Thermo Fisher Scientific; Supplementary Figure 3), using the 7500 Fast Real-Time PCR System (Applied Biosystems, Thermo Fisher Scientific). Relative quantity against control gene RNA18S5 (Hs03928985_g1) was calculated using the dCT method. XIST expression (Hs01079824_m1) was used to confirm sample identity (data not shown).

To identify the alternative transcript in P32, PCR was performed on cDNA using the Q5 High-Fidelity 2X Master Mix (New England BioLabs, Ipswich, MA, USA; M0492) with annealing temperature of 62˚C and the following primer pair specific to BCL11A-XL isoform (and other secondary isoforms) but not BCL11A-L or -S: 5’- TCTGGGCACAGGCATAGTTG-3’ (F), 5’- GTCCAAAAAGCTGCTGCTGG-3’ (R). PCR products were resolved on GelRed stained 1.2% agarose gels, and analyzed by dideoxynucleotide sequencing.

***Fetal Hemoglobin quantification***

Fetal hemoglobin was quantified independently in clinical labs by each referring center. Quantification was performed consistently using standard high-performance liquid chromatography (HPLC) methods, however we cannot control for inter-laboratory and inter-system variability.

#### Immunohistochemistry

Fetal hindbrain at 18pcw was fixed in 4% Paraformaldehyde (PFA) and transferred to methacarn (60% absolute methanol, 30% chloroform, 10% glacial acetic acid) prior to embedding in paraffin wax and subsequent sectioning. Following heat mediated antigen retrieval with citrate buffer pH 6, slides were blocked with 1% BSA overnight at 4˚C and incubated with primary antibody in 1% BSA for 1 hour at room temperature. Slides were incubated with secondary antibodies diluted in 1% BSA at 1:500 45 minutes at room temperature in the dark. Nuclei were stained with DAPI at 1:10.000 in PBS. Slides were Incubated in 0.1% Sudan Black for 20 min at room temperature and mounted with VectaMount AQ aqueous mounting medium (Vector Laboratories; Newark CA; H-5501). Slides were imaged on the Zeiss Axio Scan.Z1 microscope with the software module ZEN slidescan (ZEISS, Oberkochen, Germany). Images were further analyzed on QuPath v.0.2.3(3). Only minor adjustments limited to contrast and brightness to the entire image were performed.

Fetal hindbrain at 12pcw and 16pcw were processed and analyzed as described in ref. (4).

Antibodies used are provided in Supplementary Table 9.

***GestaltMatcher analysis***

We performed the GestaltMatcher approach(5, 6) to analyze the facial similarities among the 33 individuals with BCL11A-IDD. The cohort consisted of 34 images of 23 individuals who consented to the facial photo analysis in this paper and 12 images from ten individuals reported by the previous publications (Supplementary Table 1). We encoded each image into twelve 512-dimensional vectors using model ensemble and test-time augmentation. By computing the average cosine distance between these vectors, we quantified the facial phenotypic similarity between pairs of images. A smaller distance indicates a higher degree of facial phenotypic similarity, suggesting proximity within the phenotype space.

First, we conducted the cohort-level analysis to verify the similarity among individuals with the BCL11A gene. Next, we performed an individual-level analysis to explore these similarities further.

**Facial similarity of BCL11A-IDD**. We first calculated BCL11A-IDD individuals’ mean pairwise distance and random sampled 100 times to validate their similarities. We made sure the images from the individual were not sampled together to avoid bias. In Figure 4B, we compared the BCL11A-IDD distribution (orange) to two distributions (same and random) built from the 1,555 images from different subjects with 328 syndromes from the GestaltMatcher Database (GMDB)(7). For each of the 328 syndromes, we randomly selected a sub-cohort and computed the mean pairwise distance 100 times to build the "same" distribution (shown in blue). Additionally, we generated the "random" distribution (shown in red) by randomly sampling a sub-cohort without constraining them within the same syndrome and calculating their mean pairwise distance 100 times.

To obtain the threshold for distinguishing the same and random distributions, we then performed a five-fold cross-validation with Receiver Operating Characteristic (ROC) analysis. The threshold c was determined by the maximum Youden index, resulting in c=0.909, corresponding to a sensitivity of 0.862 and a specificity of 0.792. In the end, 34.5% of BCL11A-IDD distribution was below the threshold. It indicated that only a partial of the cohort were similar. If we only focused on the photos taken below ten years old, 58.8% of the distribution was below the threshold (Supplementary Figure 3A), suggesting the patients below ten years old showed higher similarity than the older patients.

We further utilized pairwise comparison analysis to investigate the facial similarities on the individual level. We compared 46 images of 33 individuals with BCL11A-IDD to 7,459 images with 449 different disorders from GMDB by performing the leave-one-out cross-validation to simulate the real-world scenario. We can visualize the similarity of each pair of individuals compared to the control cohort. In Supplementary Figure 4, by testing the image of P9, we put the remaining 45 images in the space with the other 7,459 images and calculated the ranks of these images to P9, and it showed that individual Balci_1_Basak_3 was at the 9th closest position to P9. Supplementary Figure 4 shows no clear cluster in general or for specific mutation types. The clusters were mainly formed by the images from the same individual. Dizygotic twins P2 and P3 ranked 1^st^ and 2^nd^ against their sibling, respectively.

To investigate the similarities among the mutation types, we further perform tSNE to project the images to two-dimensional space.(8) No clear clusters were observed (Supplementary Figure 3B).

Therefore, with the above analyses, we concluded that no clear facial gestalt was present in the BCL11A-IDD cohort, but the patients below ten years old show relatively high similarities. In addition, no clear cluster linked to specific mutation types was detected. However, this could be influenced by the limited size of the sub-cohorts with specific mutation types, requiring reanalysis with larger sub-cohorts in the future.

***Genetic analysis***

Individuals had previously been diagnosed through Next Generation Sequencing (NGS) panels, exome sequencing, genome sequencing or genomic microarray as part of local diagnostic or research studies for developmental delay, intellectual disability or autism spectrum disorder. All participants had provided informed consent for diagnostic or research testing according to local jurisdiction guidelines and relevant ethical review board requirements.

Where available, methods according to each contributor are detailed:

Individuals recruited to the Deciphering Developmental Disorders Study: per ref. (9).

Individuals recruited to the 100,000 Genomes Project and Genomics England: per ref. (10).

Patients tested by GeneDx: Using genomic DNA from the proband and parent(s), the exonic regions and flanking splice junctions of the genome were captured using the Clinical Research Exome kit (Agilent Technologies, Santa Clara, CA, USA) or the IDT xGen Exome Research Panel v1.0. Massively parallel (NextGen) sequencing was done on an Illumina system with 100bp or greater paired-end reads. Reads were aligned to human genome build GRCh37/UCSC hg19, and analyzed for sequence variants using a custom-developed analysis tool. Additional sequencing technology and variant interpretation protocol has been previously described(11). The general assertion criteria for variant classification are publicly available on the GeneDx ClinVar submission page ([http://www.ncbi.nlm.nih.gov/ clinvar/](http://www.ncbi.nlm.nih.gov/%20clinvar/)[submitters/26957/](http://www.ncbi.nlm.nih.gov/clinvar/submitters/26957/)).

Patients tested by ARUP Laboratories: Cytogenomic SNP microarray testing was performed using the CytoScan HD platform (Thermo Fisher Scientific).

C4RCD Research Group: PCR-free genome sequencing was performed at the research labs at the Translational Genomics Research Institute using the Illumina NovaSeq6000. Emedgene (https://www.emedgene.com/) software was employed for variant analysis.

Recruited by O.L.B.: Trio sequencing of the proband and the parents was performed by 150bp paired end exome sequencing on the Illumina Nextseq 500 with Nextera Rapid Capture Custom Kit. An inhouse bioinformatics pipeline was applied, using bwa alignment and GATK/Picard for variant calling. Variant annotation was performed with Annovar. For variant analysis, the DDDG2P-gene list (<https://decipher.sanger.ac.uk/info/ddg2p>) was applied to filter clinically relevant variants using the Filtus tool(12).

Recruited by A.B.: DNA-laboratory of the Department of Clinical Genetics, Erasmus MC University Medical Center, Rotterdam. Methods available on request.

Recruited by A.S.: per ref. (13)

Recruited by B.B.A.d.V: per ref. (14).

Recruited by K.A., W.B.: per ref. (15).

For additional genetics analyses methods please contact the authors.

### D. Supplementary Information – Consortia members

**C4RCD Research Group**, Center for Rare Childhood Disorders, Translational Genomics Research Institute, Phoenix, Arizona. The following members (listed in alphabetical order) have contributed significantly to this work: Newell Belnap (nbelnap@tgen.org), Anna Bonfitto (abonfitto@tgen.org), Matthew Huentelman (mhuentelman@tgen.org), Wayne Jepsen (wjepsen@tgen.org), Vinodh Narayanan (vnarayanan2@tgen.org), Marcus Naymik (mnaymik@tgen.org), Keri Ramsey (kramsey@tgen.org), Sampathkumar Rangasamy (srangasamy@tgen.org), Meredith Sanchez-Castillo(msanchez-castillo@tgen.org), and Szabolics Szelinger (sszelinger@tgen.org). This group includes the clinical team and laboratory research team involved in patient enrollment, sample processing, exome sequencing, data processing, preparation of variant annotation files, data analysis, validation of data, and return of research data to families. Candidate genes are identified and discussed at data analysis meetings of the entire group.

**Telethon Undiagnosed Diseases Program:** Vincenzo Nigro^a, b^, Annalaura Torella^a, b^, Michele Pinelli^c^, Manuela Morleo^a, b^, Carmine Spampanato^b^, Sandro Banfi^a, b^, Alessandra Varavallo^a^, Giulio Piluso^b^, Giuseppina Vitiello^c^, Angelo Selicorni^d^, Milena Mariani^d^, Marta Massimello^e^, Cecilia Daolio^e^, Valeria Capra^f^, Andrea Accogli^f^, Marcello Scala^f^, Vincenzo Leuzzi^g^, Francesca Nardecchia^g^, Serena Galosi^g^, Mario Mastrangelo^g^, Donatella Milani^h^, Corrado Romano^i^, Pinella Failla^i^, Donatella Greco^i^, Chiara Pantaleoni^j^, Claudia Ciaccio^j^, Stefano D’Arrigo^j^, Nicola Brunetti Pierri^a,k^, Giancarlo Parenti^a, k^, Antonietta Coppola^k^, Teresa Mattina^l^, Marcella Zollino^m^, Simona Amenta^m^, Albina Tummolo^n^, Claudia Santoro^o^, Anna Grandone^o^, Daniele De Brasi^p^, Antonio Varone^p^, Livia Garavelli^q^, Carla Marini^r^, Stefania Bigoni^s^, Carmelo Piscopo^t^, Antonio Trabacca^u^, Marta De Rinaldis^u^, Angela Peron^v,w,x^

^a^Telethon Institute of Genetics and Medicine, Pozzuoli, Naples, Italy; ^b^Department of Precision Medicine, University of Campania “Luigi Vanvitelli”, Naples, Italy; ^c^Department of Translational Medicine, Federico II University, Naples, Italy; ^d^Department of Pediatrics, ASST Lariana San’'Anna Hospital, San Fermo Della Battaglia, Como, Italy; ^e^MBBM Foundation, Monza, Italy; ^f^Neuroscience Department, Giannina Gaslini Institute, Genoa, Italy; ^g^Department of Human Neuroscience, Sapienza University of Rome, Italy; ^h^Pediatric Highly Intensive Care Unit, Fondazione IRCCS Ca’ Granda, Ospedale Maggiore Policlinico, Milan, Italy; ^i^Oasi Research Institute–- IRCCS, Troina, Italy; ^j^Developmental Neurology Unit, Fondazione IRCCS Istituto Neurologico Carlo Besta, Milan, Italy; ^k^Department of Translational Medicine, Section of Pediatrics, Federico II University, Naples, Italy; ^l^Department of Biomedical and Biotechnological Sciences, University of Catania, Catania, Italy; ^m^Institute of Genomic Medicine, Catholic University, Gemelli Hospital Foundation, Rome, Italy; ^n^Department of Metabolic Diseases, Clinical Genetics and Diabetology, Giovanni XXIII Children's Hospital, Bari, Italy; ^o^Pediatric Surgery, Department of Women, Children, General, and Specialist Surgery, Campania University “Luigi Vanvitelli”, Naples, Italy; ^p^Department of Pediatrics, AORN Santobono Pausilipon, Naples, Italy; ^q^Medical Genetics Unit, Azienda USL-IRCCS di Reggio Emilia, Reggio Emilia, Italy; ^r^Child Neurology and Psychiatric Unit, Pediatric Hospital G. Salesi, United Hospitals of Ancona, Ancona, Italy; ^s^Medical Genetics Unit, Department of Medical Sciences, Ferrara University, Ferrara, Italy; ^t^Azienda Ospedaliera di Rilievo Nazionale Antonio Cardarelli, Naples, Italy; ^u^IRCCS E Medea La Nostra Famiglia, Brindisi, Italy; ^v^Medical Genetics, ASST Santi Paolo e Carlo, San Paolo Hospital, Milan, Italy; ^w^*currently at*: Department of Experimental and Clinical Biomedical Sciences, Università degli Studi di Firenze, Italy; ^x^*currently at*: Medical Genetics, Meyer Children’s Hospital IRCCS, Firenze, Italy.

**University of Washington Center for Mendelian Genomics** **(UW-CMG)**: Michael J. Bamshad^a,b^, Suzanne M. Leal^c^, and Deborah A. Nickerson^a^, Peter Anderson^a^, Tamara J. Bacus^a^, Elizabeth E. Blue^a^, Kati J. Buckingham^a^, Jessica X. Chong^a^, Diana Cornejo Sánchez^c^, Colleen P. Davis^a^, Christian D. Frazar^a^, Danielle Giroux^a^, William W. Gordon^a^, Martha Horike-Pyne^a^, Jameson R. Hurless^a^, Gail P. Jarvik^a^, Eric Johanson^a^, J. Thomas Kolar^a^, Melissa P. MacMillan^a^, Colby T. Marvin^a^, Sean McGee^a^, Daniel J. McGoldrick^a^, Betselote Mekonnen^a^, Patrick M. Nielsen^a^, Karynne Patterson^a^, Benjamin Pelle^a^, Aparna Radhakrishnan^a^, Matthew A. Richardson^a^, Gwendolin T. Roote^a^, Erica L. Ryke^a^, Isabelle Schrauwen^c^, Kathryn M. Shively^a^, Joshua D. Smith^a^, Monica Tackett^a^, Machiko S. Threlkeld^a^, Gao Wang^c^, Jeffrey M. Weiss^a^, Marsha M. Wheeler^a^, Qian Yi^a^, Jordan E. Zeiger^a^, and Xiaohong Zhang^a^.

^a^University of Washington, ^b^Seattle Children’s Hospital, ^c^Columbia University

The full list of current members of University of Washington Center for Mendelian Genomics is available at http://uwcmg.org/docs/Crediting_UW-CMG/UW_CMG_Banner.pdf.

### E. Supplementary Reference List

1. Karczewski KJ, Francioli LC, Tiao G, Cummings BB, Alföldi J, Wang Q, et al. The mutational constraint spectrum quantified from variation in 141,456 humans. Nature. 2020;581(7809):434-43.

2. Yang Y, Xu Z, He C, Zhang B, Shi Y, Li F. Structural insights into the recognition of γ-globin gene promoter by BCL11A. Cell Res. 2019;29(11):960-3.

3. Bankhead P, Loughrey MB, Fernández JA, Dombrowski Y, McArt DG, Dunne PD, et al. QuPath: Open source software for digital pathology image analysis. Scientific Reports. 2017;7(1):16878.

4. Haldipur P, Aldinger KA, Bernardo S, Deng M, Timms AE, Overman LM, et al. Spatiotemporal expansion of primary progenitor zones in the developing human cerebellum. Science. 2019;366(6464):454-60.

5. Hsieh T-C, Bar-Haim A, Moosa S, Ehmke N, Gripp KW, Pantel JT, et al. GestaltMatcher facilitates rare disease matching using facial phenotype descriptors. Nature Genetics. 2022;54(3):349-57.

6. Hustinx A, Hellmann F, Sumer O, Javanmardi B, Andre E, Krawitz P, et al. Improving Deep Facial Phenotyping for Ultra-rare Disorder Verification Using Model Ensembles. 2023 IEEE/CVF Winter Conference on Applications of Computer Vision (WACV): IEEE; 2023.

7. Lesmann H, Hustinx A, Moosa S, Marchi E, Caro P, Abdelrazek IM, et al. GestaltMatcher Database - A global reference for the facial phenotypic variability of rare human diseases. medRxiv. 2024:2023.06.06.23290887.

8. van der Maaten L, Hinton G. Visualizing Data using t-SNE. Journal of Machine Learning Research. 2008;9(86):2579-605.

9. The Deciphering Developmental Disorders S. Large-scale discovery of novel genetic causes of developmental disorders. Nature. 2015;519(7542):223-8.

10. Genomics England Research C. Sample Handling Guidance. version 4.0 ed2018.

11. Retterer K, Juusola J, Cho MT, Vitazka P, Millan F, Gibellini F, et al. Clinical application of whole-exome sequencing across clinical indications. Genetics in Medicine. 2016;18(7):696-704.

12. Vigeland MD, Gjøtterud KS, Selmer KK. FILTUS: a desktop GUI for fast and efficient detection of disease-causing variants, including a novel autozygosity detector. Bioinformatics. 2016;32(10):1592-4.

13. Beleford DT, Van Ziffle J, Hodoglugil U, Slavotinek AM. A missense variant, p.(Ile269Asn), in MC4R as a secondary finding in a child with BCL11A-related intellectual disability. European Journal of Medical Genetics. 2020;63(9):103969.

14. Lelieveld SH, Reijnders MRF, Pfundt R, Yntema HG, Kamsteeg E-J, de Vries P, et al. Meta-analysis of 2,104 trios provides support for 10 new genes for intellectual disability. Nature Neuroscience. 2016;19(9):1194-6.

15. Aldinger KA, Timms AE, Thomson Z, Mirzaa GM, Bennett JT, Rosenberg AB, et al. Redefining the Etiologic Landscape of Cerebellar Malformations. The American Journal of Human Genetics. 2019;105(3):606-15.
